# Supplementary material for: Evidence for single metal two electron oxidative addition and reductive elimination at uranium
Source: Nat Commun. 2017 Dec 1;8:1898. doi: 10.1038/s41467-017-01363-0 (PMC5711956; doi:10.1038/s41467-017-01363-0)
Supplement: Supplementary file 1 — Supplementary Information [file 41467_2017_1363_MOESM1_ESM.pdf]

Supplementary Figures

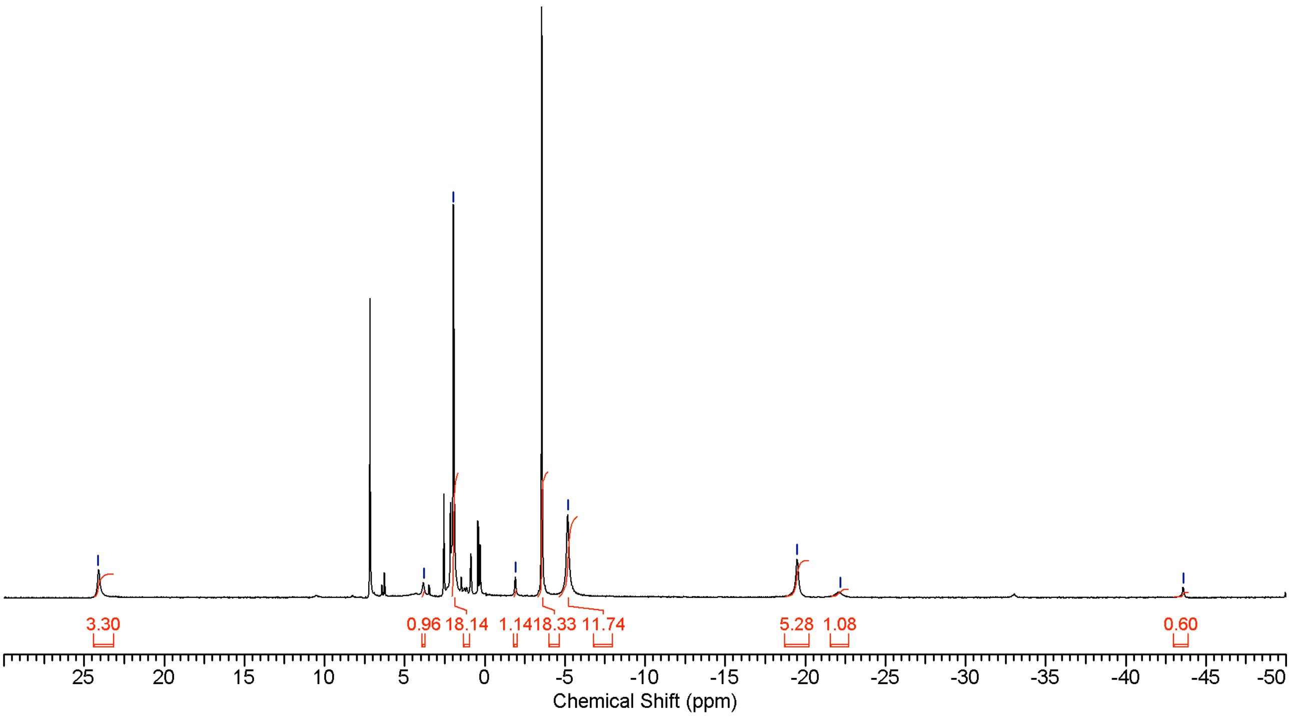

Supplementary Figure 1. ¹H NMR spectrum of 2.tmeda.

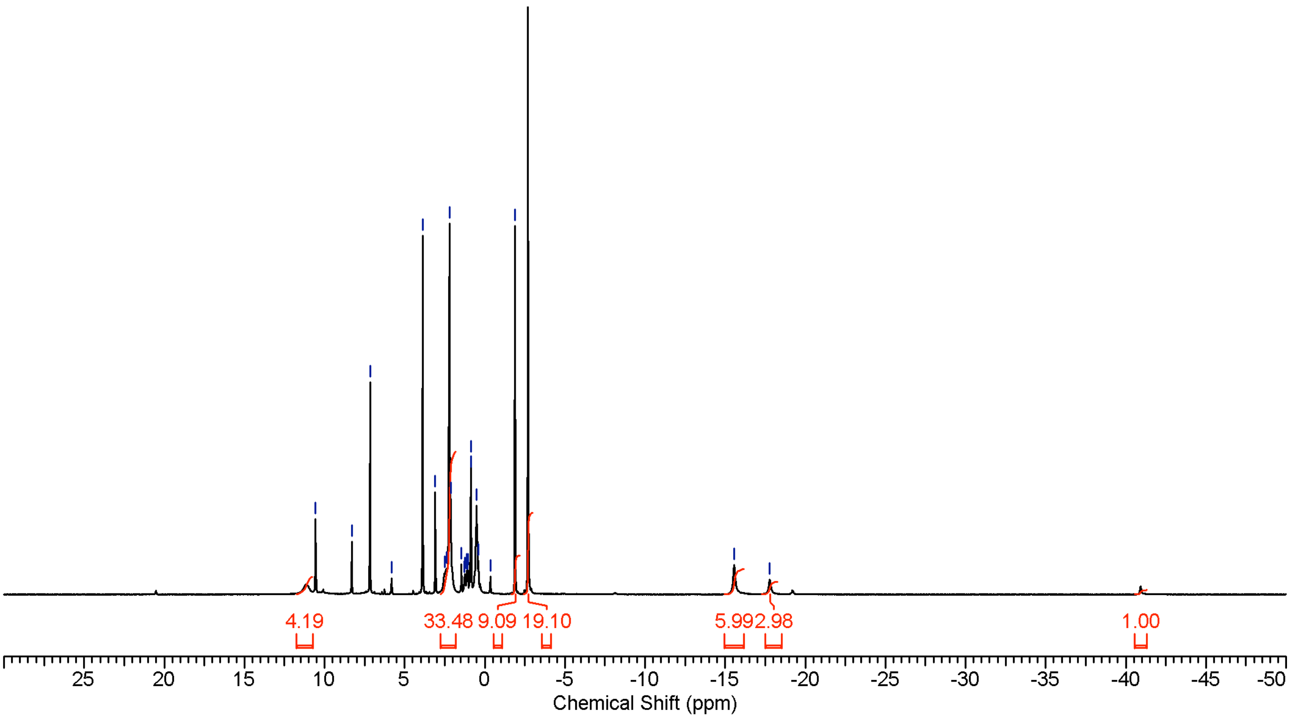

Supplementary Figure 2. ¹H NMR spectrum of 2.pmdeta.

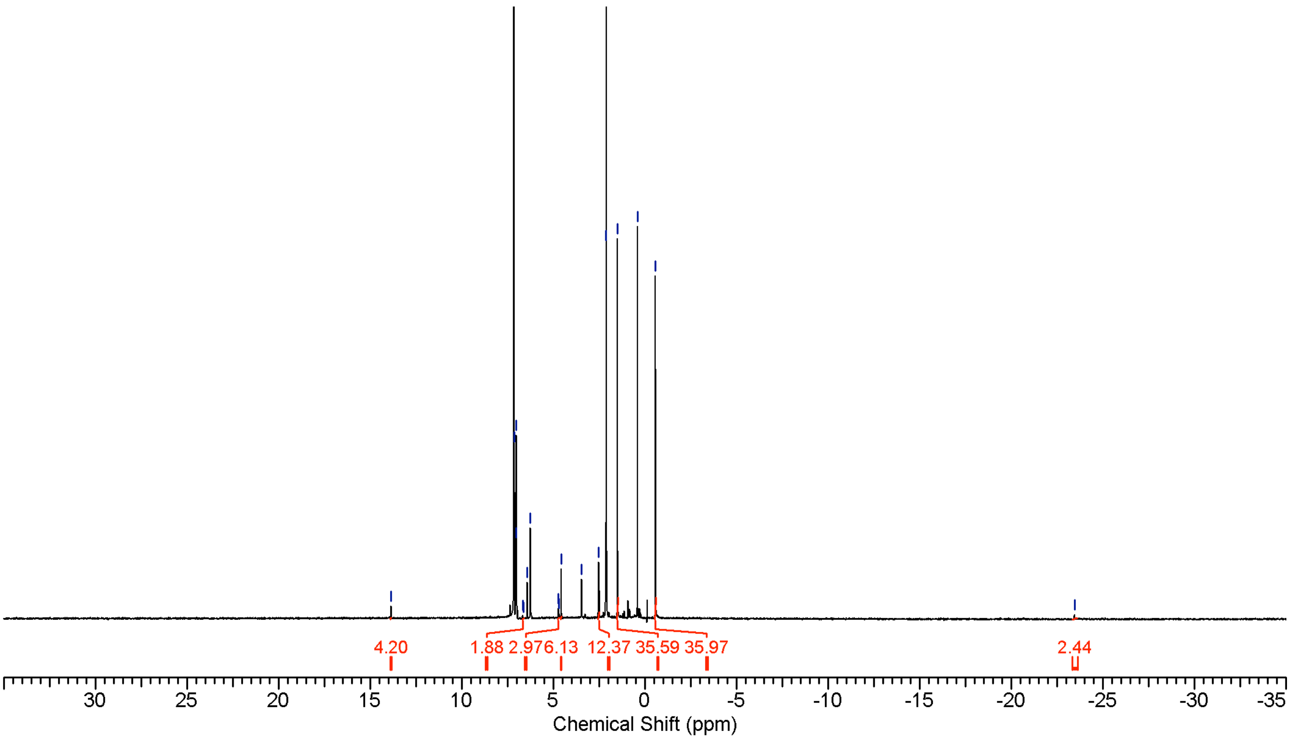

Supplementary Figure 3. ***^{1}H NMR spectrum of 3.***

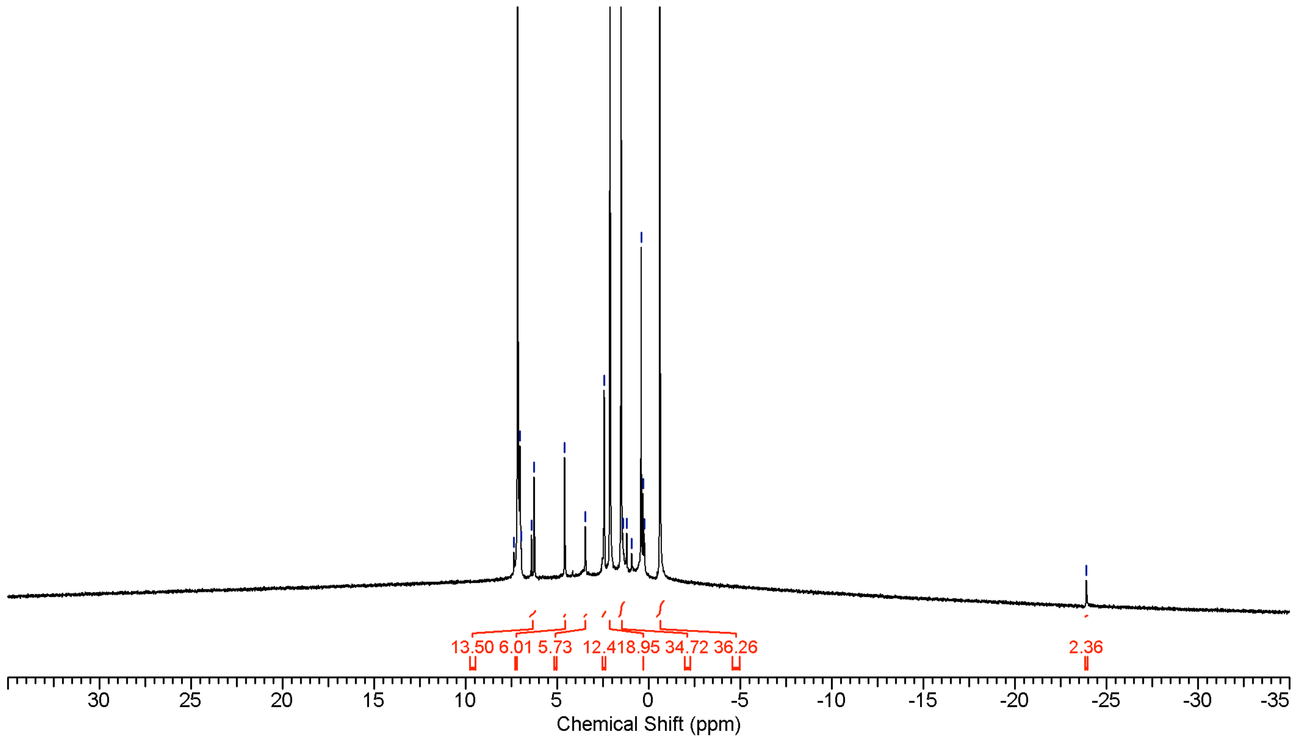

Supplementary Figure 4. ^{1}H NMR spectrum of 3-D_{10}.

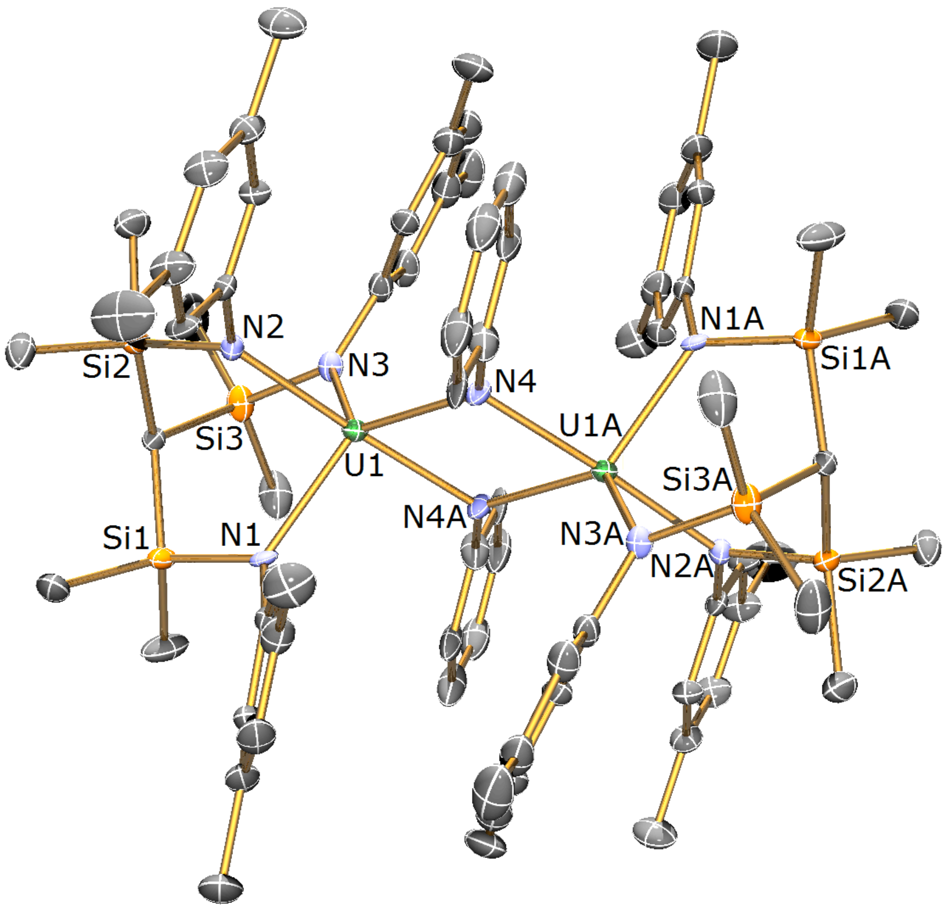

Supplementary Figure 5. Molecular structure of [{U(Ts^{Xy})(μ-NPh-D$_5$)}$_2$] (3-D$_{10}$) at 90 K with 40% probability ellipsoids. *Hydrogen/deuterium atoms are omitted for clarity. Selected distances: U1-N1 2.209(7), U1-N2 2.221(6), U1-N3 2.230(6), U1-N4 2.196(7), U1-N4A 2.198(6) Å.*

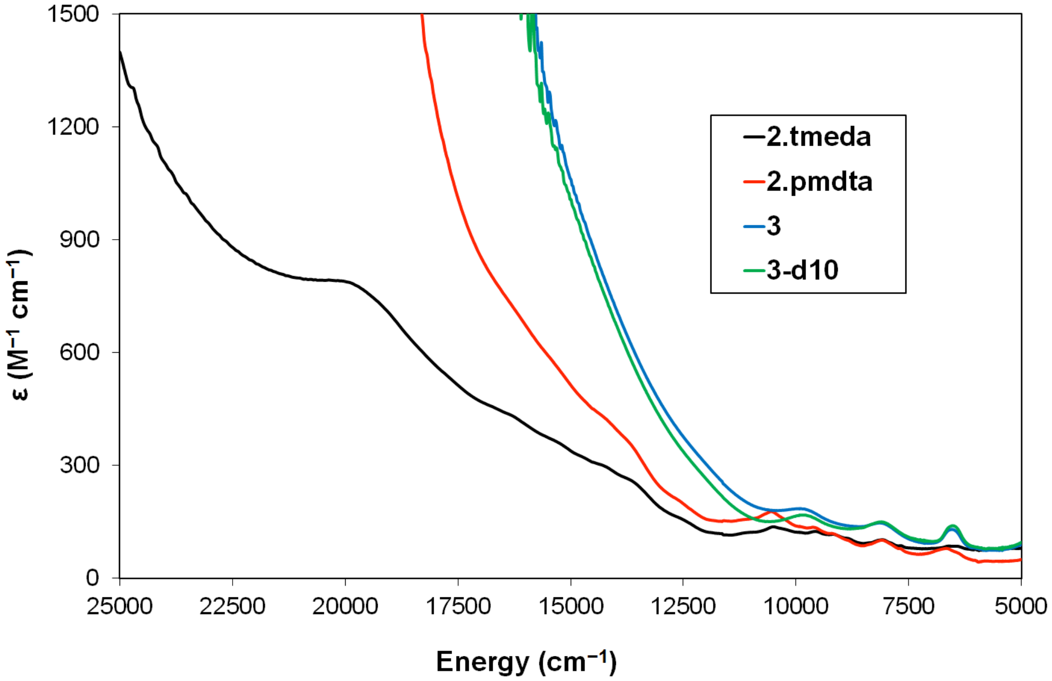

Supplementary Figure 6. UV/Vis/NIR spectrum of 2.tmeda, 2.pmdeta, 3, and 3-D$_{10}$.

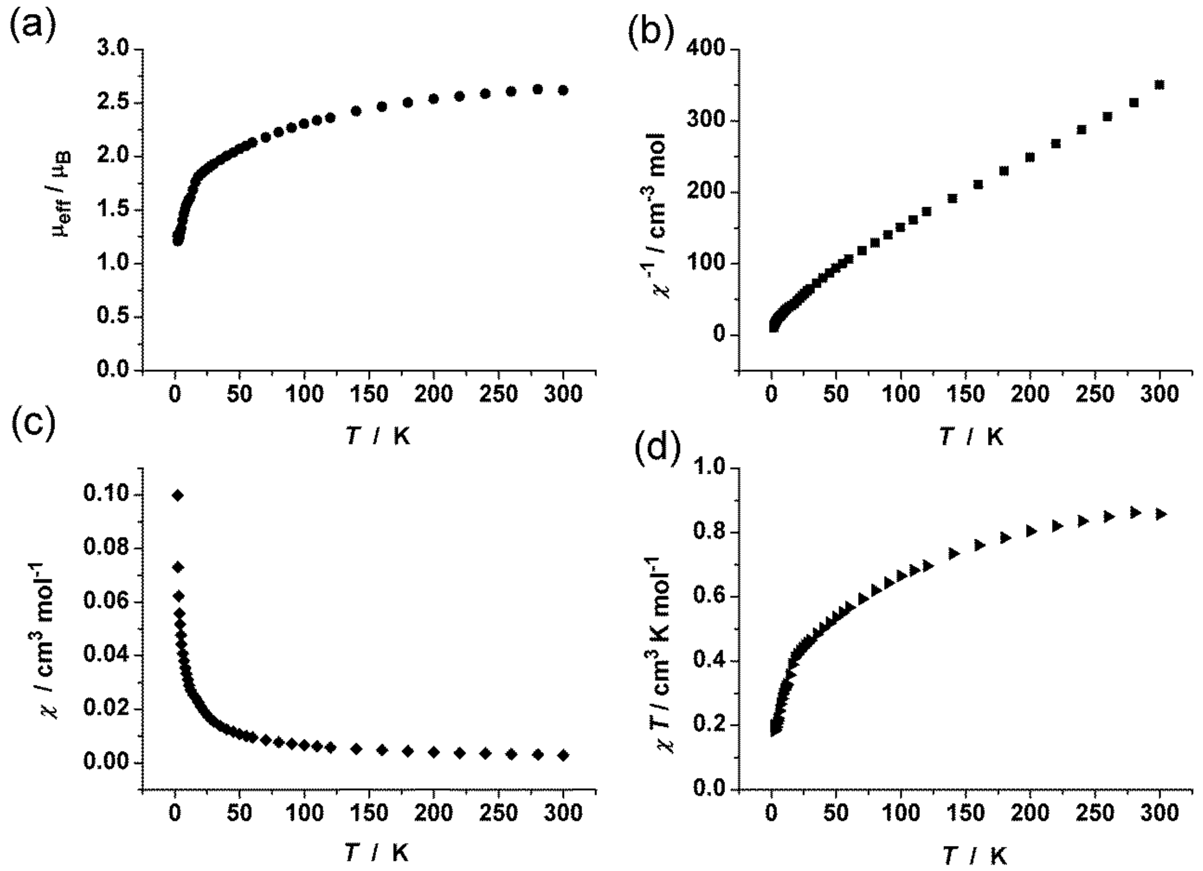

Supplementary Figure 7. Magnetic data for 2.tmeda as a function of temperature (T) measured on a powder sample, measured in a 0.1 T applied magnetic field. *(a) effective magnetic moment (μ_{eff}), (b) inverse molar magnetic susceptibility (χ^{-1}), (c) molar magnetic susceptibility (χ), and (d) product of magnetic susceptibility and temperature (χT).*

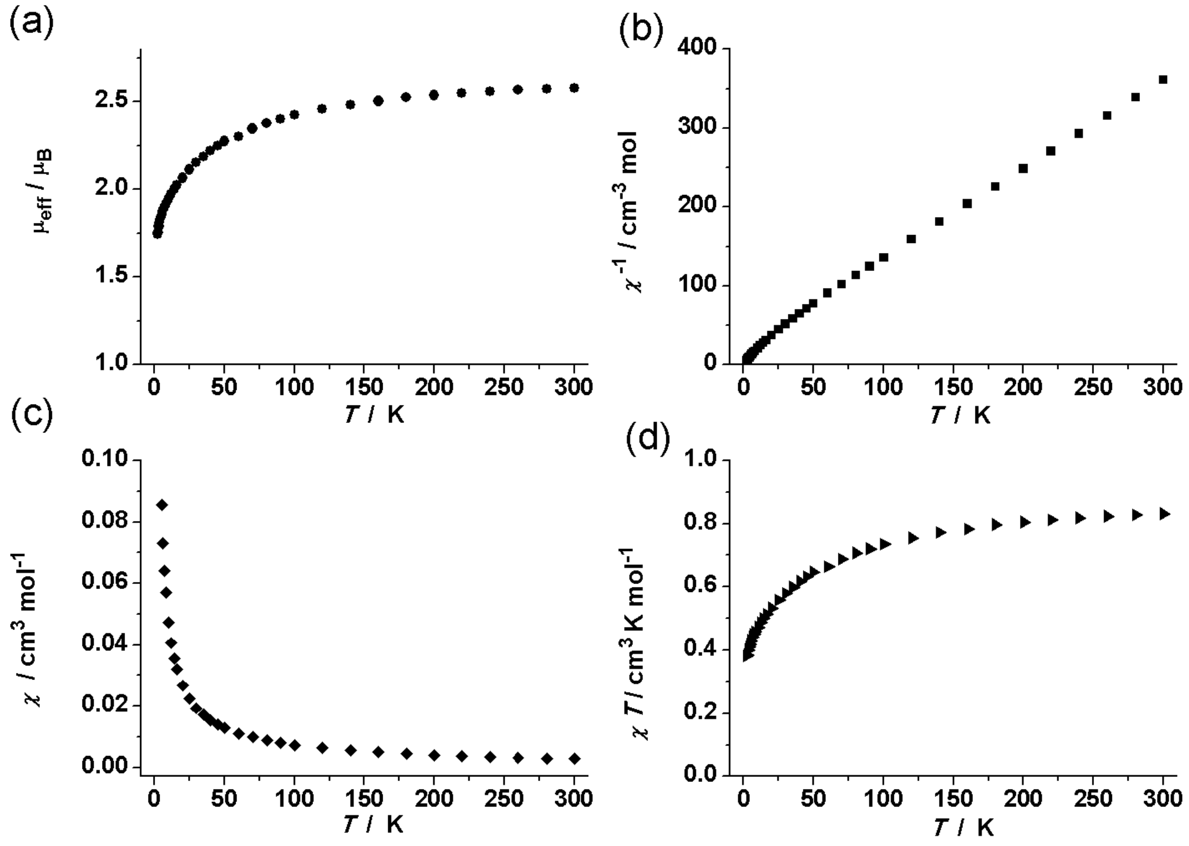

Supplementary Figure 8. Magnetic data for 2.pmdeta as a function of temperature (T) measured on a powder sample, measured in a 0.1 T applied magnetic field. *(a) effective magnetic moment (μ_{eff}), (b) inverse molar magnetic susceptibility (χ^{-1}), (c) molar magnetic susceptibility (χ), and (d) product of magnetic susceptibility and temperature (χT).*

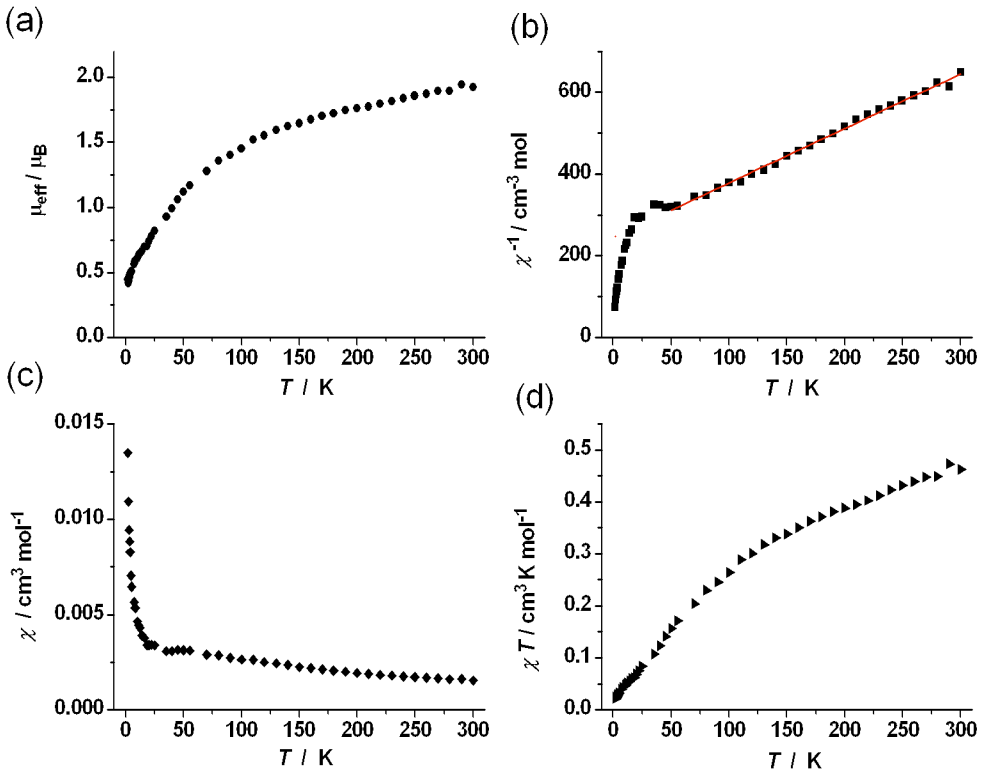

Supplementary Figure 9. Magnetic data for 3 (powder sample), measured in a 0.1 T applied magnetic field, data are per dimer. *The limiting data in (a) correspond to 1.36* μ_B *per uranium ion at 298 K, falling to 0.31* μ_B *per uranium ion at 2 K. The red line in (b) is a fit of the 298 – 50 K data to a Curie-Weiss equation,* $\chi = C/(T-\theta)$*, giving a Curie constant* $C = 1.73$ μ_B *(per U) and a Weiss temperature* $\theta = -184$ *K. For comparison we list the limiting effective magnetic moments (per U) reported for other uranium(V) dimers with a* $\{U_2X_2\}$ *bridging core where* X^{2-} *is a dianionic bridging ligand: (i) 1.52 → 0.37* μ_B *(300 to 2 K) in* $[\{(^{Ad}ArO)_3N\}UO\}]_2$ $((^{Ad}ArO)_3N^{3-}$ *= trianion of tris(2-hydroxy-3-adamantyl-5-methylbenzyl)amine) (30);*[1] *(ii) 1.5 → 0.2* μ_B *(300 to 2 K) in* $[(Me_3SiOUO)_2L]$ *(Curie constant from high temperature data,* $C = 1.57$ μ_B *per U;* L^{4-} *= "pacman" Schiff-base polypyrrolic macrocycle);*[2] *(iii) 1.69 → 0.3* μ_B *(300 to 2 K) in* $[UO_2(dbm)_2K(18C6)]_2$ *(Weiss temperature from high T data,* $\theta = -195$ *K;* dbm^- *= anion of dibenzoylmethane , 18C6 = 18-crown-6 ether).*[3] *Uranium(V) dimers with bridging imides have been reported,*[4] *but these involve {U(N-Ar-N)U} cores so are not directly comparable.*

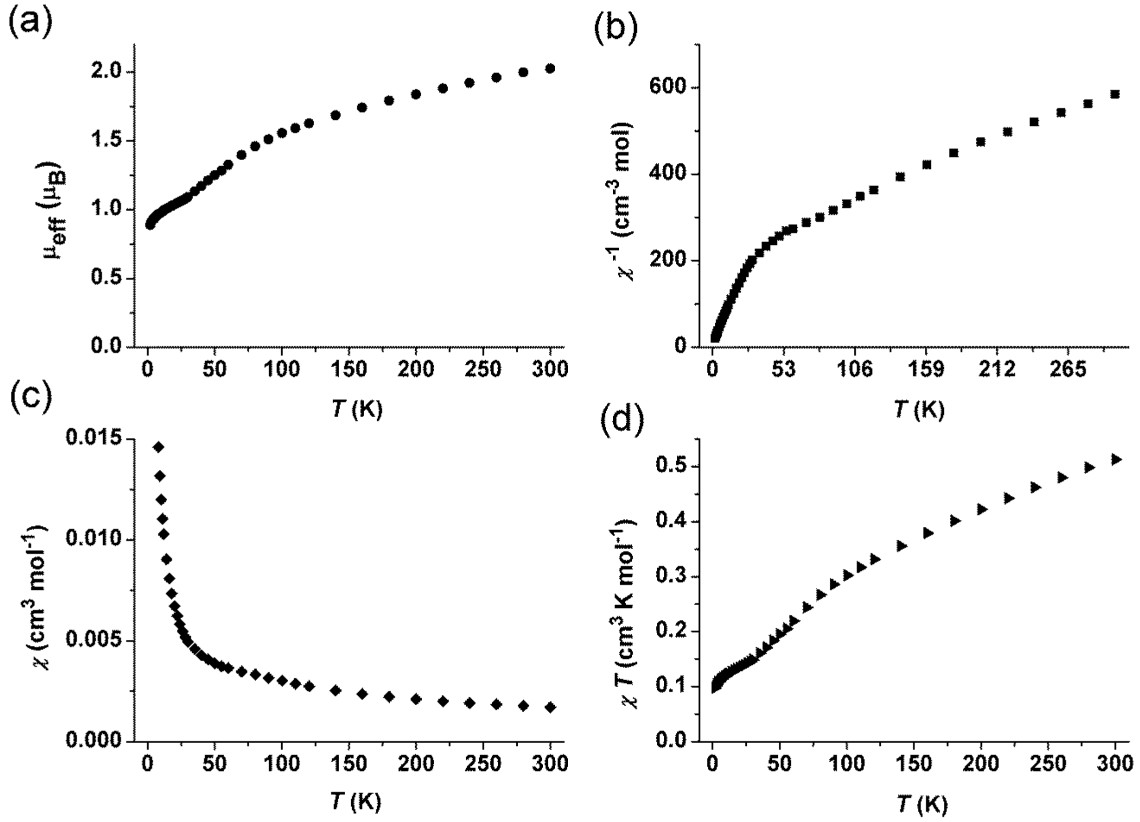

Supplementary Figure 10. Magnetic data for 3-D_{10} (powder sample), measured in a 0.1 T applied magnetic field.

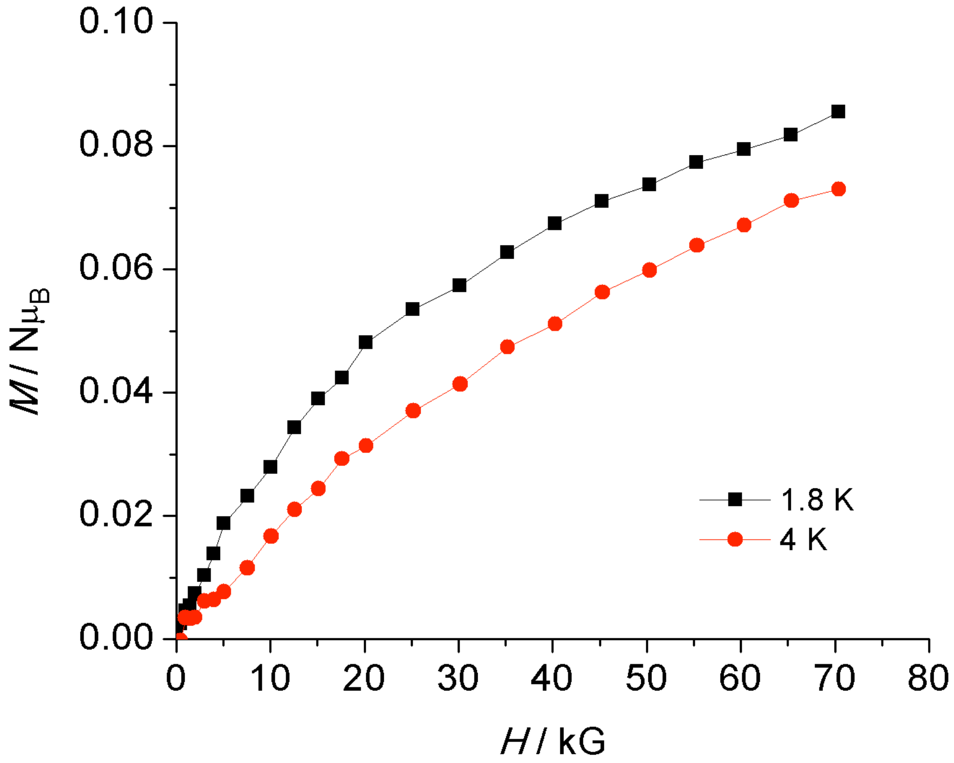

Supplementary Figure 11. Molar magnetization (M) vs applied magnetic field (H) data for 3 (powder sample) at 1.8 and 4 K.

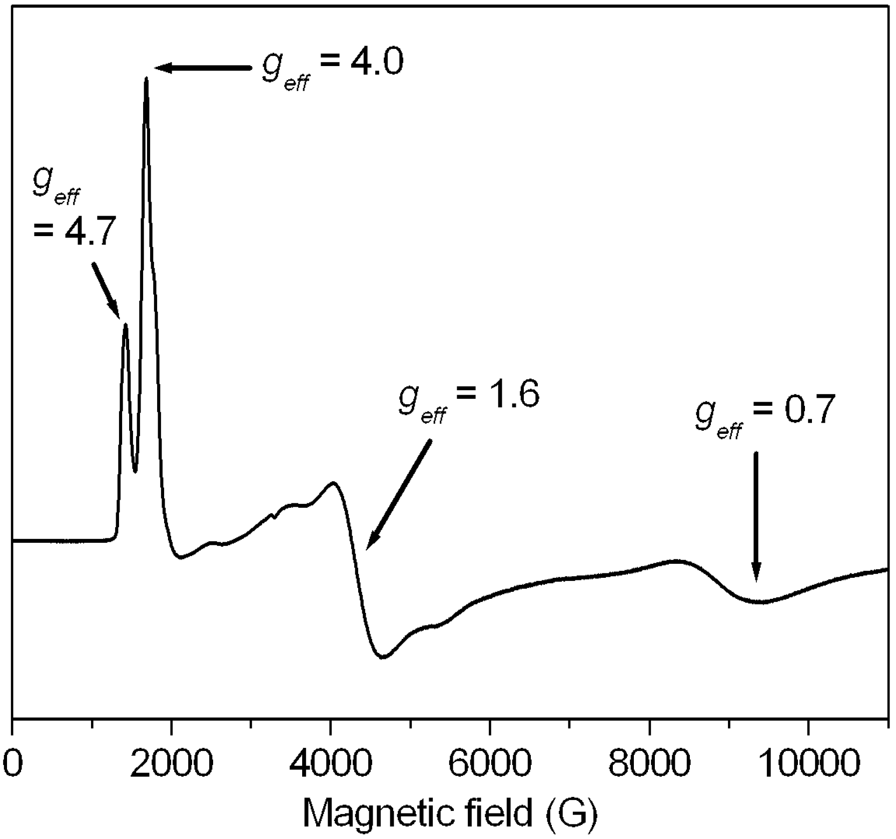

Supplementary Figure 12. X-band (9.4 GHz) EPR spectrum of a powder sample of 2.pmdeta at 4 K, with effective g-values marked. *[There is a second, minor uranium species, giving g_{eff} = 4.7, the identity of which is unknown.] For comparison, the well-characterized and related uranium(III) complex [U{N(CH$_2$CH$_2$NSiPr$^i{}_3$)$_3$}] has g_{eff} = 3.9, 1.8, 0.6.*[5]

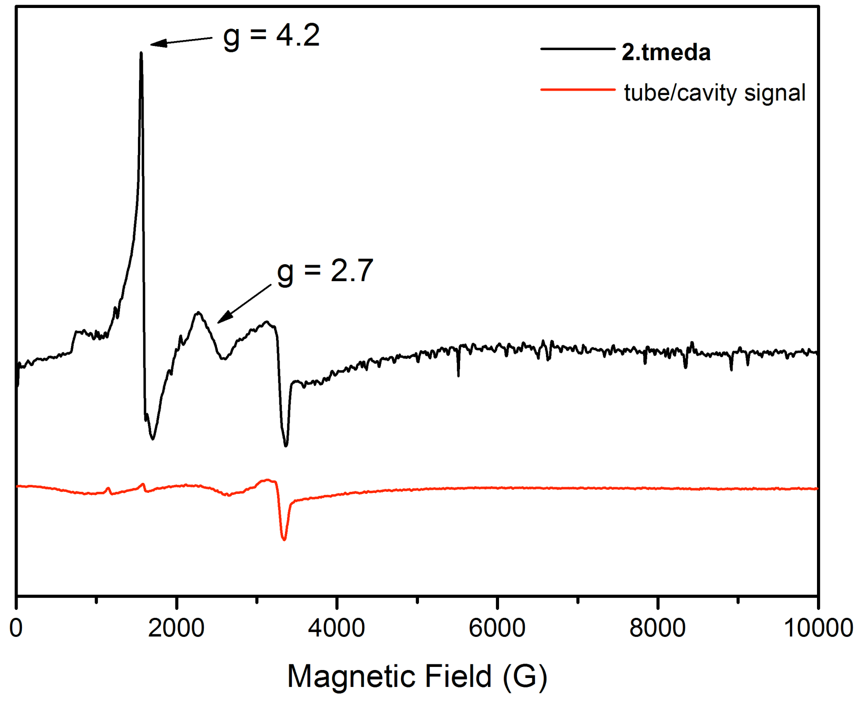

Supplementary Figure 13. X-band EPR spectrum of 2.tmeda at 5 K along with blank cavity for comparison.

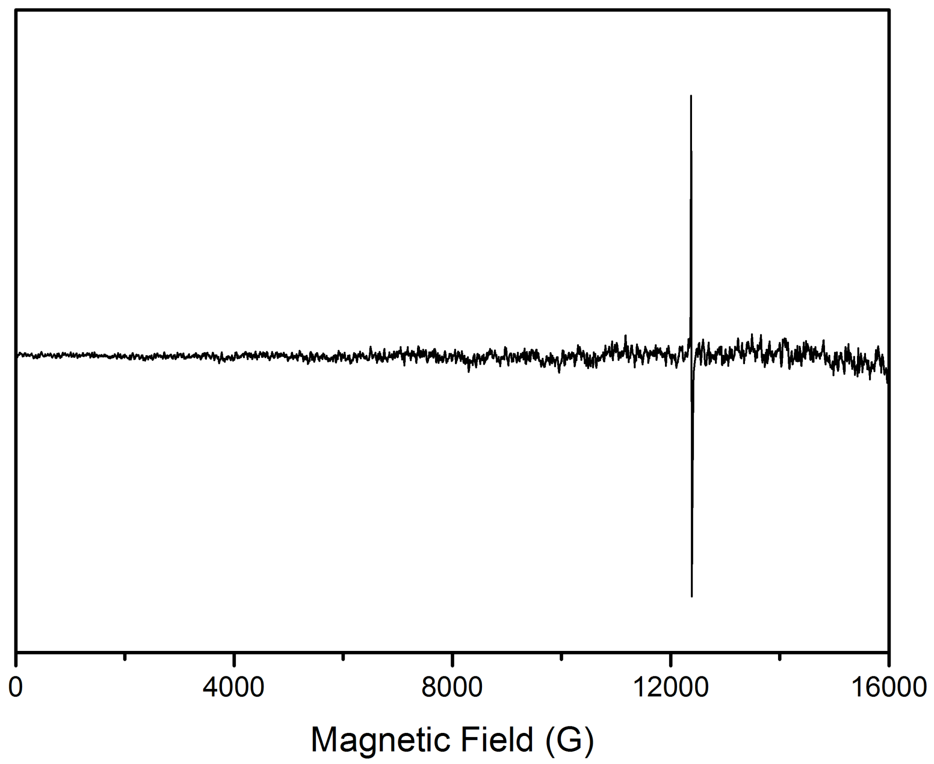

Supplementary Figure 14. Q-band (35 GHz) EPR spectrum of a powder sample of 3 at 5 K Spectra of 3-D_{10} are identical.

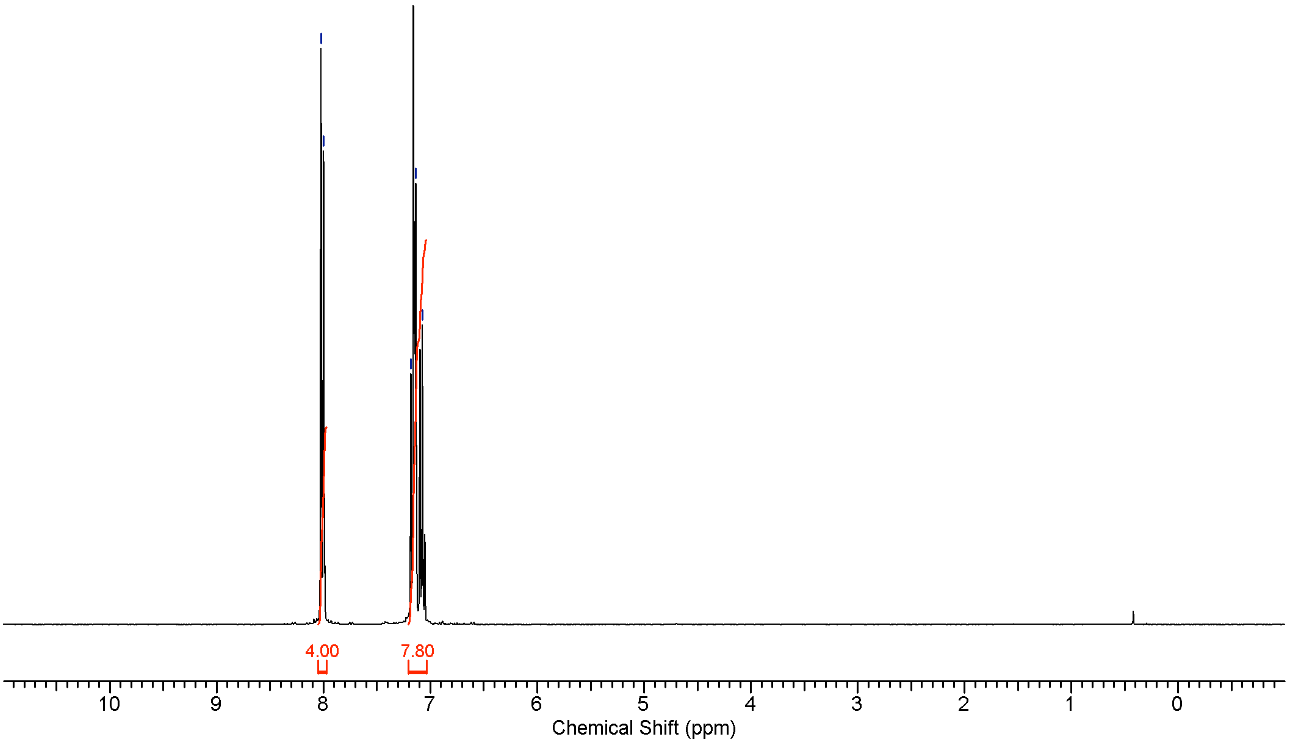

Supplementary Figure 15. ***^{1}H NMR spectrum of genuine sample of PhNNPh from a commercial supplier.***

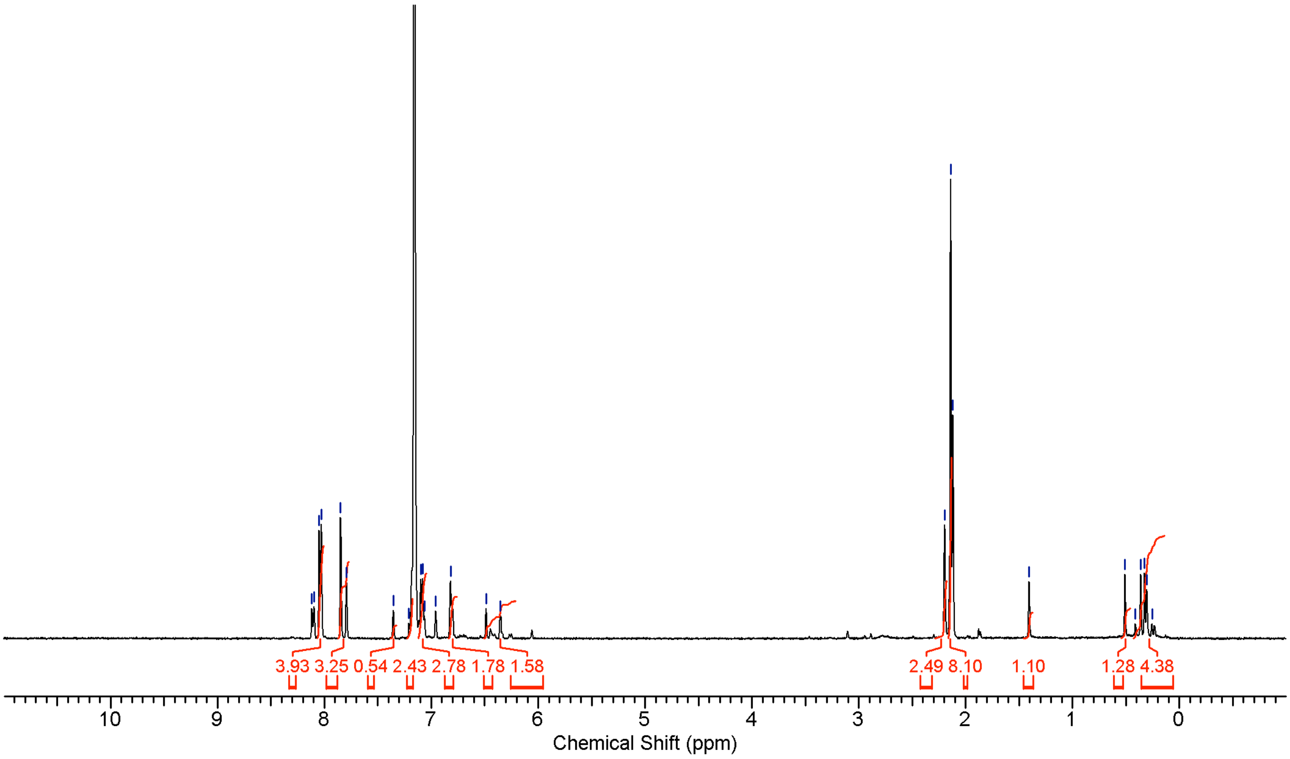

Supplementary Figure 16. ***^{1}H NMR spectrum of PhNNPh isolated from the thermally instigated reductive elimination of 3.***

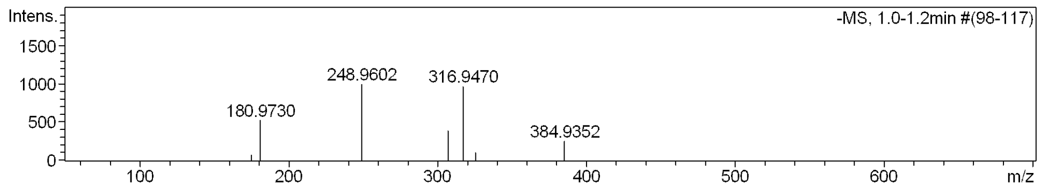

Supplementary Figure 17. Mass spectrum (ESI, negative) of genuine sample of PhNNPh from a commercial supplier. *m/z 180.97 (64%) {PhNNPh-H^+}$^-$, 248.96 (98%) {PhNNPh-H^++HCO_2Na}$^-$, 316.95 (100%) {PhNNPh-H^++2HCO_2Na}$^-$.*

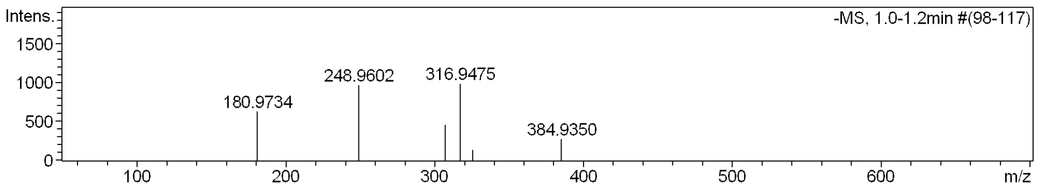

Supplementary Figure 18. Mass spectrum (ESI, negative) of PhNNPh isolated from the thermally instigated reductive elimination of 3. *m/z 180.97 (64%) {PhNNPh-H^+}$^-$, 248.96 (98%) {PhNNPh-H^++HCO_2Na}$^-$, 316.95 (100%) {PhNNPh-H^++2HCO_2Na}$^-$.*

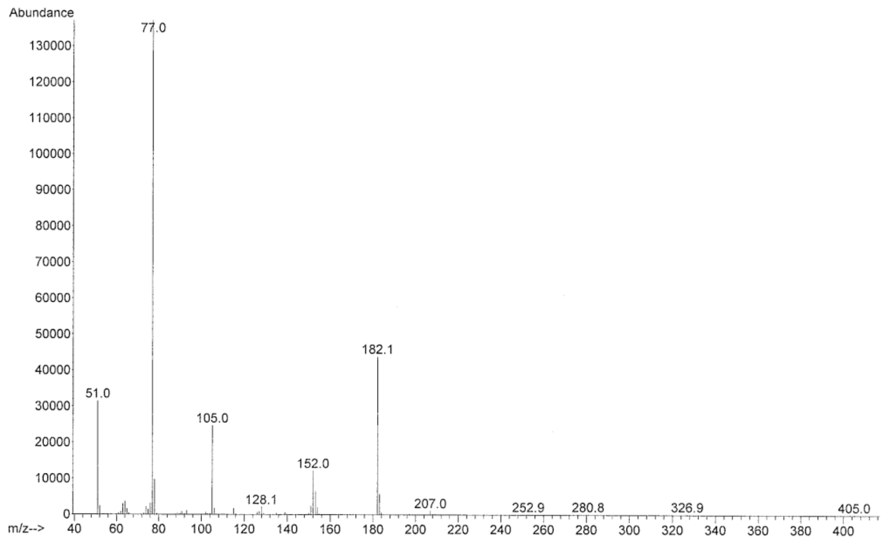

Supplementary Figure 19. Mass spectrum (ESI, positive) of PhNNPh isolated from the thermally instigated reductive elimination of 3. *m/z 182.1 (32%) {PhNNPh}$^+$.*

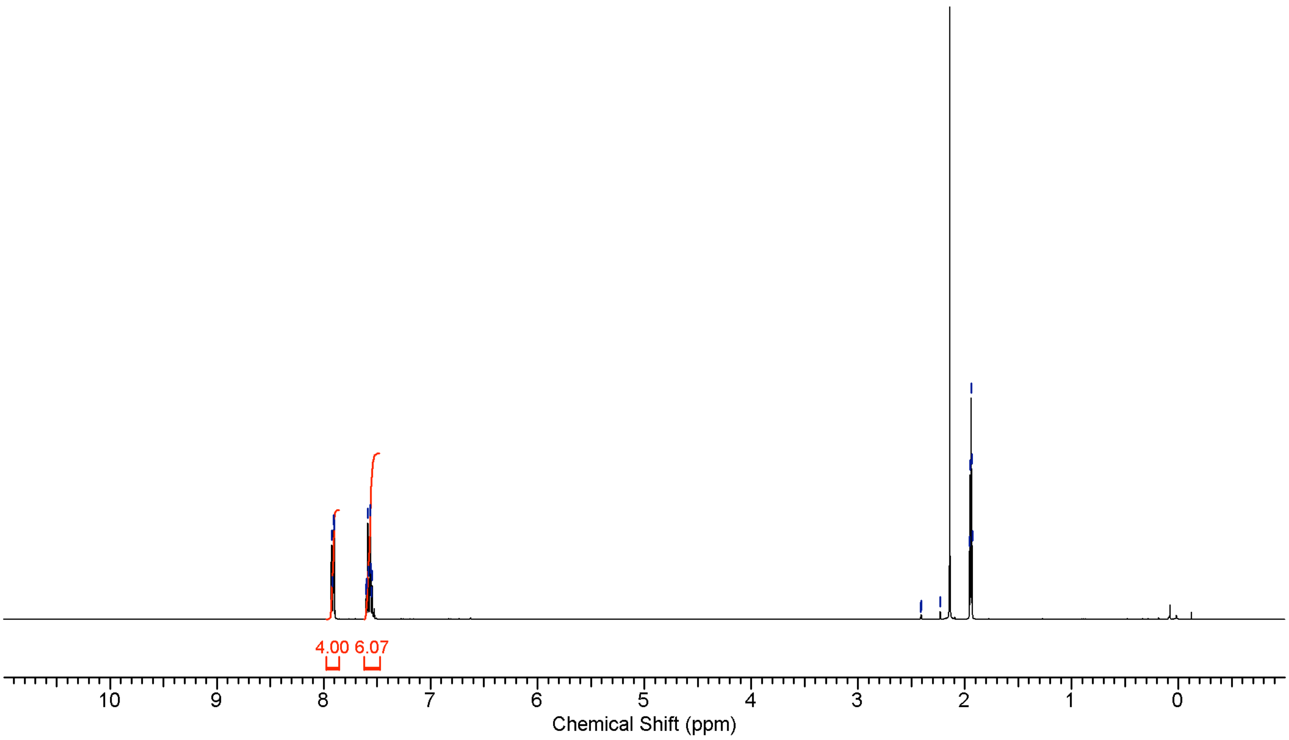

Supplementary Figure 20. ***^{1}H NMR spectrum (in CD_3CN) of PhNNPh/D_{10}-PhNNPh mixture isolated from the thermally instigated reductive elimination of an equimolar mixture of 3/3-D_{10}.***

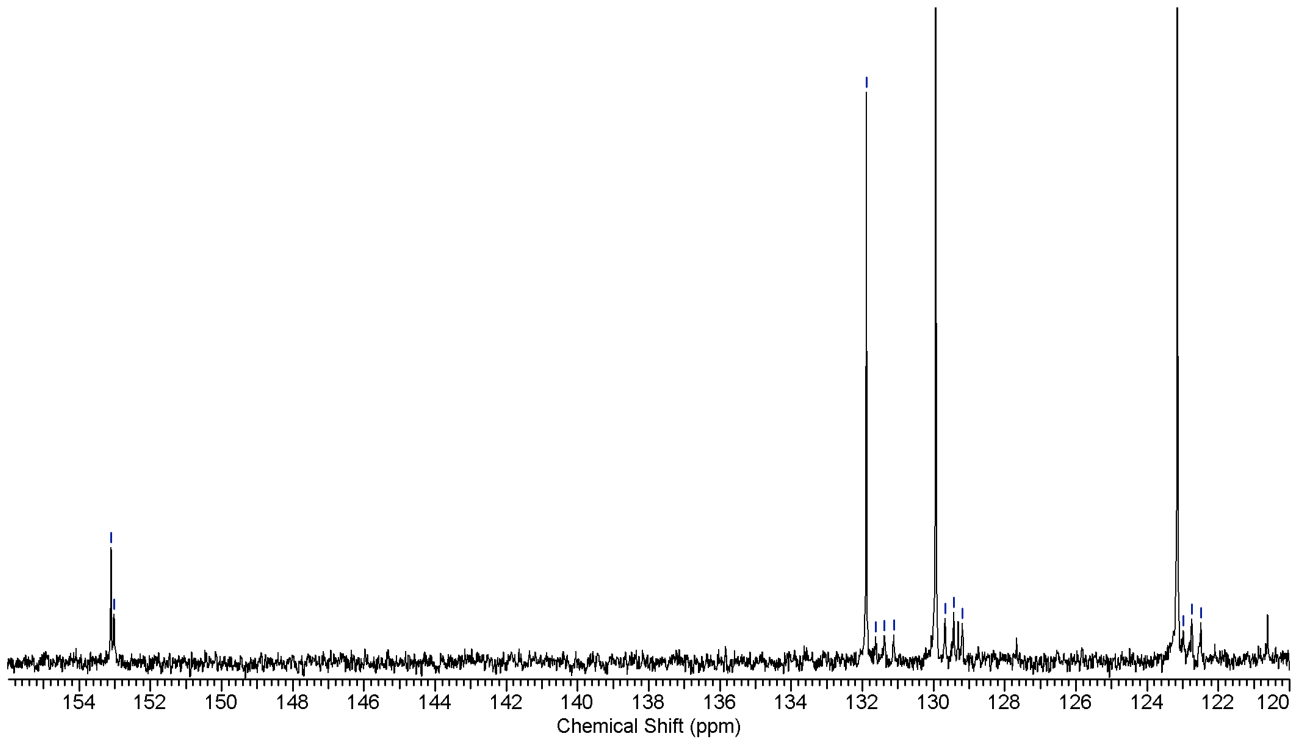

Supplementary Figure 21. ***$^{13}C\{^1H\}$ NMR spectrum (in CD_3CN) of PhNNPh/D_{10}-PhNNPh mixture isolated from the thermally instigated reductive elimination of an equimolar mixture of 3/3-D_{10}.***

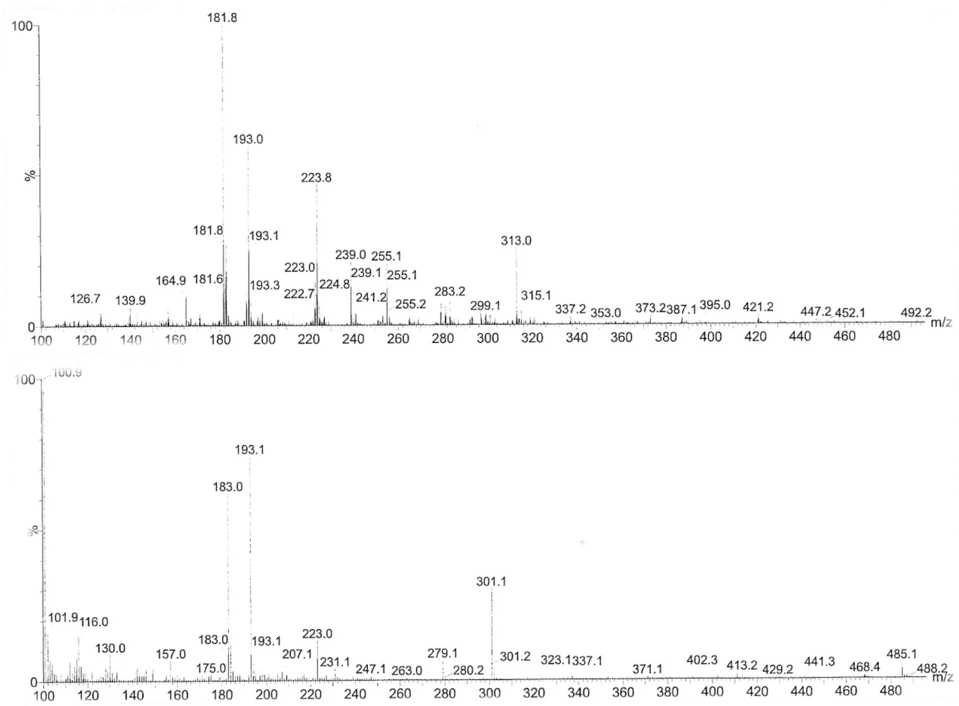

Supplementary Figure 22. Mass spectra of PhNNPh/PhNNPh-D_{10} mixture isolated from the thermally instigated reductive elimination of a 50:50 mixture of 3/3-D_{10}. *Top (ESI negative, MeOH) m/z 181.8 (100%) {PhNNPh−H^+}$^-$, 193.0 (60%) {PhNNPh-D_{10}+H}$^-$, 223.8 (45%) {PhNNPh-D_{10}+MeO^-}$^-$. Bottom (ESI positive, MeOH): m/z 183.0 (62%) {PhNNPh+H^+}$^+$, 193.1 (75%) {PhNNPh-D_{10}+H^+}$^+$.*

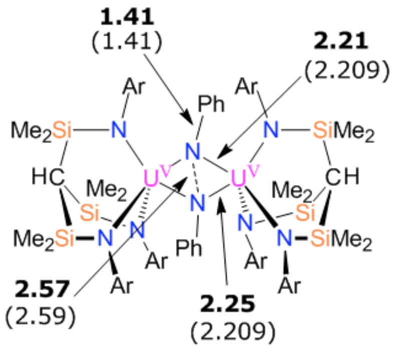

Supplementary Figure 23. Selected bond distances of complex 3 in angstroms. *The values in parenthesis correspond to those found in the X-ray available structure.*

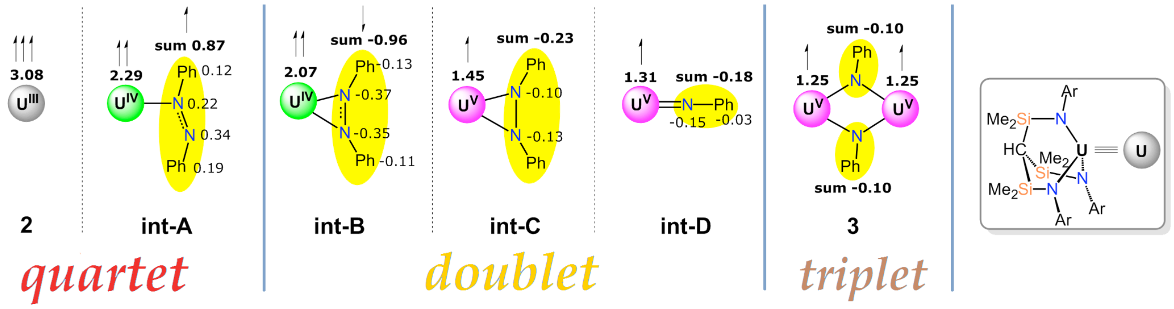

Supplementary Figure 24. Calculated spin densities for the key intermediates and complexes reported in this work.

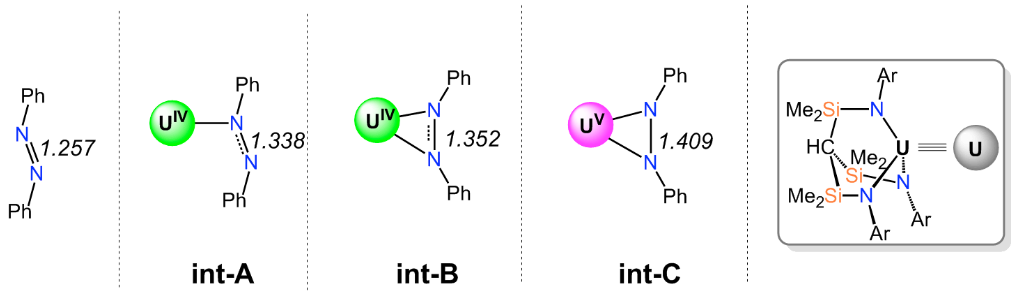

Supplementary Figure 25. Calculated N-N bond distances for key intermediates in this work in angstroms.

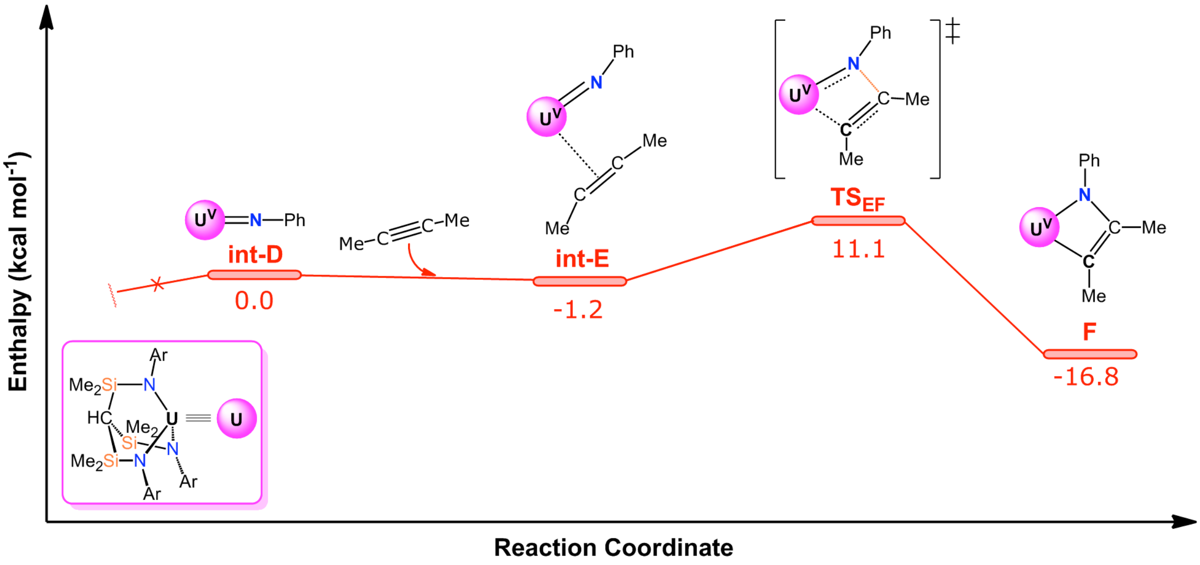

Supplementary Figure 26. Computed enthalpy profile of the reaction of putative intermediate int-D with 2-butyne to afford the insertion product F.

Supplementary Tables

Supplementary Table 1. Experimental X-ray crystallographic details for 2.tmeda, 2.pmdeta, 3, and 3-D$_{10}$. CCDC deposition numbers 1529405 – 1529408.

	**2.tmeda**	**2.pmdeta•C$_6$H$_{14}$**	**3•2C$_6$H$_{14}$**	**3-D$_{10}$•2C$_7$H$_8$**
Formula	$C_{37}H_6N_5Si_3U$	$C_{46}H_{83}N_6Si_3U$	$C_{86}H_{130}N_8Si_6U_2$	$C_{88}H_{108}D_{10}N_8Si_6U_2$
Fw	899.21	1042.48	1920.57	1942.56
Cryst size, mm^3	0.29 x 0.13 x 0.08	0.57 x 0.25 x 0.09	0.29 x 0.10 x 0.02	0.16 x 0.13 x 0.10
Cryst syst	Orthorhombic	Orthorhombic	Triclinic	Triclinic
Space group	*P*na2$_1$	*P*bca	*P*-1	*P*-1
a, Å	28.2456(3)	18.1705(8)	13.3083(5)	13.2627(6)
b, Å	11.18817(9)	20.8800(19)	13.4052(5)	13.4687(6)
c, Å	13.45048(13)	27.2577(15)	14.7536(6)	14.7640(10)
α, °	90	90	111.114(4)	111.040(5)
β, °	90	90	114.087(4)	113.116(5)
γ, °	90	90	95.390(3)	96.914(4)
V, Å^3	4250.57(7)	10341.6(12)	2149.96(17)	2156.4(2)
Z	4	8	1	1
ρ_{calc} g cm-^{3}	1.405	1.339	1.483	1.496
μ, mm^{-1}	11.776	9.758	11.676	3.882
no. of reflections measd	92658	25851	22850	14578
no. of unique reflns, Rint	8615, 0.0826	10201, 0.0483	8472, 0.0578	7585, 0.0682
no. of reflns with $F^2 > 2s(F^2)$	8253	8651	7891	6273
transmn coeff range	0.23 – 0.67	0.40-0.97	0.18-0.92	0.66-0.76
R, R_w^{a} ($F^2 > 2s(F^2)$)	0.0356, 0.0931	0.0557, 0.1505	0.0380, 0.0979	0.0553, 0.1178
R, R_w^{a} (all data)	0.0371, 0.0949	0.0670, 0.1572	0.0410, 0.1001	0.0709, 0.1297
S^a	1.034	1.079	1.073	1.048
Parameters	581	496	418	602
max.,min. diff map, e Å^{-3}	2.846, -1.955	2.224, -5.368	1.678, -1.536	2.484, -2.502

Computer programs: *CrysAlis PRO*, Agilent Technologies, Version 1.171.36.32 (release 02-08-2013 CrysAlis171 .NET) (compiled Aug 2 2013,16:46:58); *CrysAlis PRO*, Agilent Technologies, Version 1.171.35.11 (release 16-05-2011 CrysAlis171 .NET) (compiled May 16 2011,17:55:39), *SHELXTL, SHELXL*, and *SHELXL97* (Sheldrick, 2008), Olex2 (Dolomanov *et al.*, 2009), *enCIFer*(Allen *et al.*, 2004), *PLATON* (Spek, 2009).

Supplementary Table 2. Selected bond lengths (Å) and angles (°) for 2. tmeda, 2.pmdeta, 3 and 3-D₁₀.

**2.tmeda**			
U1-N1	2.304(7)	U1-N2	2.302(8)
U1-N3	2.320(7)	U1-N4	2.770(8)
U1-N5	2.757(7)	Si1-N1	1.719(8)
Si1-C1	1.888(10)	Si1-C2	1.884(10)
Si1-C3	1.883(10)	Si2-N2	1.724(8)
Si2-C1	1.877(10)	Si2-C12	1.877(13)
Si2-C13	1.886(11)	Si3-N3	1.722(8)
Si3-C1	1.903(9)	Si3-C22	1.862(12)
Si3-C23	1.888(12)	N1-C4	1.403(9)
N2-C14	1.380(10)	N2-C14A	1.461(11)
N3-C24	1.406(9)	N3-C24A	1.419(12)
N4-C32	1.468(15)	N4-C33	1.455(15)
N4-C34	1.405(15)	N5-C35	1.465(14)
N5-C36	1.437(17)	N5-C37	1.452(15)
N1-U1-N3	88.2(3)	N1-U1-N4	92.8(2)
N1-U1-N5	133.4(3)	N2-U1-N1	94.3(3)
N2-U1-N3	91.7(3)	N2-U1-N4	98.9(3)
N2-U1-N5	127.7(2)	N3-U1-N4	169.3(3)
N3-U1-N5	107.2(2)	N5-U1-N4	64.7(2)
**2.pmdeta•C_6H_{14}**			
U1-N1	2.373(5)	U1-N2	2.394(6)
U1-N3	2.355(5)	U1-N4	2.832(7)
U1-N5	2.866(7)	U1-N6	2.898(6)
Si1-N1	1.731(6)	Si1-C1	1.889(6)
Si1-C2	1.885(8)	Si1-C3	1.884(8)
Si2-N2	1.706(6)	Si2-C1	1.892(6)
Si2-C12	1.891(9)	Si2-C13	1.880(7)
Si3-N3	1.731(6)	Si3-C1	1.868(7)
Si3-C22	1.891(7)	Si3-C23	1.888(7)
N1-C4	1.419(8)	N2-C14	1.390(9)
N3-C24	1.390(8)	N4-C32	1.468(11)
N4-C33	1.464(12)	N4-C34	1.466(12)
N5-C35	1.485(13)	N5-C36	1.442(14)
N5-C37	1.493(13)	N6-C38	1.467(13)
N6-C39	1.486(12)	N6-C40	1.467(12)
N1-U1-N2	85.81(19)	N1-U1-N4	92.87(19)
N1-U1-N5	102.9(2)	N1-U1-N6	154.1(2)
N2-U1-N4	170.88(19)	N2-U1-N5	125.7(2)
N2-U1-N6	88.1(2)	N3-U1-N1	94.57(19)
N3-U1-N2	88.2(2)	N3-U1-N4	82.90(19)
N3-U1-N5	142.3(2)	N3-U1-N6	110.3(2)
N4-U1-N5	63.4(2)	N4-U1-N6	96.9(2)

N5-U1-N6	61.3(2)		
**$3 \bullet 2C_6H_{14}$**			
U1-N1	2.221(4)	U1-N2	2.227(4)
U1-N3	2.203(4)	U1-N4	2.209(4)
U1-N4	2.210(4)	Si1-N1	1.755(4)
Si1-C1	1.876(5)	Si1-C2	1.870(7)
Si1-C3	1.880(6)	Si2-N2	1.746(5)
Si2-C1	1.894(5)	Si2-C12	1.860(7)
Si2-C13	1.863(7)	Si3-N3	1.750(5)
Si3-C1	1.925(5)	Si3-C22	1.868(7)
Si3-C23	1.881(6)	N1-C4	1.414(7)
N2-C14	1.414(7)	N3-C24	1.423(7)
N4-C32	1.403(7)		
N1-U1-N2	107.30(17)	N3-U1-N1	88.45(16)
N3-U1-N2	88.29(17)	N3-U1-N4a	104.45(17)
N3-U1-N4	176.15(15)	N4-U1-N1	94.22(16)
N4-U1-N1a	128.74(16)	N4-U1-N2	93.54(17)
N4-U1-N2a	122.21(17)	N4-U1-N4	71.72(19)
**$3\text{-}D_{10} \bullet 2C_7H_8$**			
U1-N1	2.209(7)	U1-N2	2.221(6)
U1-N3	2.230(6)	U1-N4a	2.198(6)
U1-N4	2.196(7)	Si1-N1	1.746(6)
Si1-C1	1.887(7)	Si1-C2	1.873(9)
Si1-C3	1.862(9)	Si2-N2	1.746(7)
Si2-C1	1.866(8)	Si2-C12	1.884(10)
Si2-C13	1.861(9)	Si3-N3	1.725(7)
Si3-C1	1.904(8)	Si3-C22	1.875(11)
Si3-C23	1.875(10)	N1-C4	1.447(9)
N2-C14	1.423(9)	N3-C24	1.431(10)
N4-C32A	1.428(14)	N4-C32	1.436(15)
N1-U1-N2	88.0(2)	N1-U1-N3	88.3(2)
N2-U1-N3	106.9(2)	N4-U1-N1	176.4(2)
N4-U1-N1	105.8(2)	N4-U1-N2	94.4(2)
N4a-U1-N2	128.3(2)	N4-U1-N3	93.6(2)
N4a-U1-N3	122.7(2)	N4a-U1-N4	70.6(3)

Supplementary Table 3. Computed Data for 2

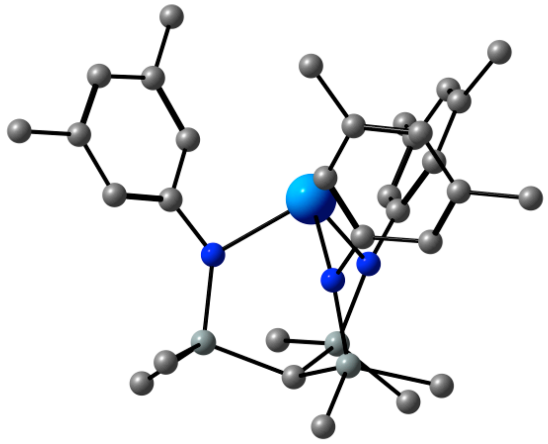

Temperature 298.150 Kelvin. Pressure 1.00000 Atm.

Zero-point correction= 0.694341 (Hartree/Particle)
Thermal correction to Energy= 0.742720
Thermal correction to Enthalpy= 0.743664
Thermal correction to Gibbs Free Energy= 0.606386
Sum of electronic and zero-point Energies= -1861.143199
Sum of electronic and thermal Energies= -1861.094820
Sum of electronic and thermal Enthalpies= -1861.093876
Sum of electronic and thermal Free Energies= -1861.231154

Charge = 0 Multiplicity = 4

92	15.132309000	13.054596000	10.366599000
7	15.841072000	13.628910000	12.467629000
7	15.311643000	15.252910000	9.745258000
7	17.311310000	12.725106000	9.735508000
14	16.823417000	15.072658000	12.726701000
14	16.885118000	16.049786000	9.687379000
14	18.629932000	13.529994000	10.587434000
6	17.849982000	15.201222000	11.111765000
1	18.695728000	15.868732000	11.344728000
6	17.927368000	14.886207000	14.261389000
1	18.538848000	13.982849000	14.246466000
1	17.296061000	14.851478000	15.156632000
1	18.591790000	15.752490000	14.361644000
6	15.814341000	16.647371000	13.078806000
1	15.386656000	16.607072000	14.086667000
1	15.003241000	16.786813000	12.359689000
1	16.463713000	17.529195000	13.044923000
6	14.772837000	13.080061000	13.143854000
6	14.463661000	11.717295000	12.879268000
1	15.228830000	11.099634000	12.400419000
6	13.305328000	11.098441000	13.386021000
6	13.054875000	9.635553000	13.126055000
1	13.725792000	9.012090000	13.729498000

1	13.230815000	9.371766000	12.077662000
1	12.028328000	9.355728000	13.378352000
6	12.439992000	11.844779000	14.175773000
1	11.542729000	11.379654000	14.578872000
6	12.722913000	13.189799000	14.487158000
6	11.781020000	13.963006000	15.373513000
1	11.769768000	13.548764000	16.388668000
1	10.752708000	13.916459000	14.997703000
1	12.067570000	15.015501000	15.445327000
6	13.861841000	13.791605000	13.969898000
1	14.060984000	14.836666000	14.184326000
6	16.720074000	17.924132000	9.951900000
1	16.165819000	18.185683000	10.854425000
1	16.196489000	18.363728000	9.095269000
1	17.710250000	18.391681000	10.006463000
6	17.765418000	15.899217000	8.006954000
1	17.268629000	16.528406000	7.260176000
1	17.778897000	14.868945000	7.642435000
1	18.798986000	16.254388000	8.084419000
6	14.210348000	15.278106000	8.917300000
6	12.980013000	14.807197000	9.453098000
1	12.888128000	14.730470000	10.540479000
6	11.831372000	14.635610000	8.657443000
6	10.539093000	14.181983000	9.285525000
1	10.112840000	14.971552000	9.916080000
1	10.684256000	13.305423000	9.926339000
1	9.794822000	13.926474000	8.526293000
6	11.902140000	14.948357000	7.306096000
1	11.021418000	14.827838000	6.678714000
6	13.091842000	15.450337000	6.740952000
6	13.127282000	15.809360000	5.277928000
1	12.828986000	14.959646000	4.653279000
1	14.125922000	16.124493000	4.964236000
1	12.432027000	16.627980000	5.057913000
6	14.221304000	15.599635000	7.533526000
1	15.144657000	15.957283000	7.089196000
6	19.318017000	12.543650000	12.062542000
1	19.903207000	11.688321000	11.706898000
1	18.526411000	12.174478000	12.719743000
1	19.996625000	13.168452000	12.653858000
6	20.138769000	13.770217000	9.458539000
1	19.904988000	14.288283000	8.527422000
1	20.557679000	12.790451000	9.202295000
1	20.919948000	14.334312000	9.981542000
6	17.143173000	11.447485000	9.246798000
6	17.805706000	10.284516000	9.723178000
1	18.588244000	10.398177000	10.466653000
6	17.462622000	9.015492000	9.277349000
6	18.189676000	7.799050000	9.789555000
1	18.919197000	8.060286000	10.560649000

1	18.725584000	7.291962000	8.978561000
1	17.492024000	7.069844000	10.216801000
6	16.418288000	8.860738000	8.343756000
1	16.149912000	7.863272000	8.001789000
6	15.748217000	9.965516000	7.833730000
6	14.693853000	9.812597000	6.768237000
1	15.087188000	10.101936000	5.786100000
1	13.823141000	10.447607000	6.963372000
1	14.349280000	8.777449000	6.693809000
6	16.116249000	11.247438000	8.283592000
1	15.714395000	12.125499000	7.769961000

Supplementary Table 4. Computed Data for int-A

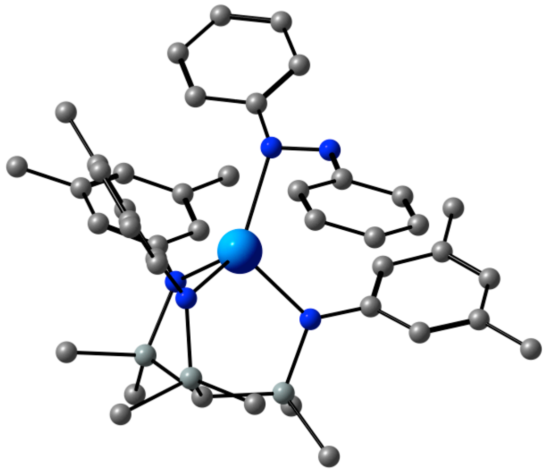

Temperature 298.150 Kelvin. Pressure 1.00000 Atm.

Zero-point correction= 0.886415 (Hartree/Particle)
Thermal correction to Energy= 0.947570
Thermal correction to Enthalpy= 0.948514
Thermal correction to Gibbs Free Energy= 0.780898
Sum of electronic and zero-point Energies= -2433.538430
Sum of electronic and thermal Energies= -2433.477275
Sum of electronic and thermal Enthalpies= -2433.476331
Sum of electronic and thermal Free Energies= -2433.643947

Charge = 0 Multiplicity = 4

92	2.334762000	8.733557000	8.785452000
14	0.267868000	8.835421000	11.478129000
14	0.122318000	6.264085000	9.544956000
14	2.639006000	6.717842000	11.542791000
7	0.746906000	9.691730000	9.988536000
7	1.133756000	6.939386000	8.239675000
7	3.363442000	8.065975000	10.645921000
7	3.054656000	10.072721000	6.900866000
6	0.778950000	7.008705000	11.185912000
6	1.079745000	9.611256000	13.009062000
6	-1.593160000	9.009478000	11.796282000
6	0.149598000	10.927663000	9.654400000
6	0.357348000	12.086164000	10.426212000
6	-0.239931000	13.300874000	10.086219000
6	-0.053141000	14.518032000	10.955835000
6	-1.035656000	13.365457000	8.936412000
6	-1.246470000	12.240742000	8.135899000
6	-2.062718000	12.334674000	6.873851000
6	-0.661695000	11.028673000	8.513222000
6	-1.685780000	6.694603000	9.171017000
6	0.138925000	4.368126000	9.655579000
6	0.840183000	6.505230000	6.923500000

6	0.274940000	7.389216000	5.994186000
6	0.008975000	6.999318000	4.676244000
6	-0.588847000	7.982143000	3.703173000
6	0.295354000	5.688559000	4.294053000
6	0.847368000	4.772706000	5.200385000
6	1.125622000	3.353807000	4.773705000
6	1.124476000	5.193248000	6.499623000
6	3.306045000	5.046557000	10.942373000
6	3.040129000	6.773305000	13.394313000
6	4.561852000	8.738685000	10.884694000
6	5.771524000	8.089451000	11.213161000
6	6.956890000	8.802268000	11.367603000
6	8.237550000	8.099979000	11.738565000
6	6.952701000	10.192527000	11.167306000
6	5.779674000	10.871945000	10.848215000
6	5.773096000	12.365204000	10.649890000
6	4.589433000	10.138939000	10.731031000
7	3.227143000	9.152246000	5.945936000
6	2.662157000	11.334854000	6.438531000
6	2.450685000	11.617901000	5.076007000
6	2.513056000	12.366561000	7.380429000
6	2.080319000	12.898204000	4.683666000
6	2.143785000	13.643522000	6.975271000
6	1.922776000	13.918913000	5.624870000
6	3.988975000	8.072045000	6.327782000
6	4.844356000	8.050320000	7.464617000
6	3.949769000	6.919401000	5.501871000
6	5.598107000	6.909913000	7.762628000
6	4.704925000	5.805735000	5.813463000
6	5.528738000	5.783793000	6.952220000

Supplementary Table 5. Computed Data for int-B

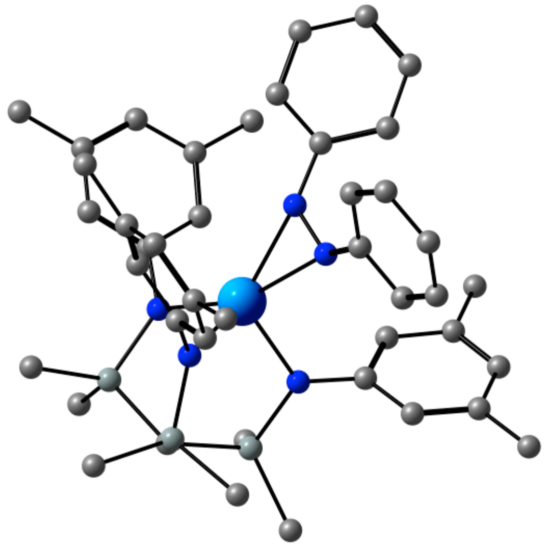

Temperature 298.150 Kelvin. Pressure 1.00000 Atm.

Zero-point correction= 0.886606 (Hartree/Particle)
Thermal correction to Energy= 0.946921
Thermal correction to Enthalpy= 0.947865
Thermal correction to Gibbs Free Energy= 0.782820
Sum of electronic and zero-point Energies= -2433.556532
Sum of electronic and thermal Energies= -2433.496217
Sum of electronic and thermal Enthalpies= -2433.495273
Sum of electronic and thermal Free Energies= -2433.660318

Charge = 0 Multiplicity = 2

92	1.711615000	8.802935000	9.024622000
14	-0.124177000	8.641586000	11.943744000
14	-0.331722000	6.234936000	9.822113000
14	2.361521000	6.646729000	11.579088000
7	0.152664000	9.553140000	10.457251000
7	0.585637000	6.981031000	8.486882000
7	2.992942000	8.009232000	10.635383000
7	2.726164000	10.063606000	7.360663000
6	0.458534000	6.879557000	11.453546000
6	0.802517000	9.373455000	13.433185000
6	-1.943925000	8.677961000	12.475677000
6	-0.160841000	10.862598000	10.102415000
6	0.054457000	11.981940000	10.938767000
6	-0.203422000	13.275079000	10.495501000
6	-0.004341000	14.456954000	11.409081000
6	-0.660876000	13.472761000	9.181646000
6	-0.875909000	12.398938000	8.320743000
6	-1.362011000	12.615416000	6.912265000
6	-0.633462000	11.101843000	8.795769000
6	-2.164715000	6.673712000	9.610224000

6	-0.287641000	4.339425000	9.758859000
6	0.363970000	6.546510000	7.159583000
6	-0.474113000	7.267852000	6.298040000
6	-0.658617000	6.883181000	4.963494000
6	-1.548010000	7.695181000	4.057437000
6	-0.004670000	5.744031000	4.496949000
6	0.830094000	4.990108000	5.334423000
6	1.528534000	3.764019000	4.804500000
6	1.006263000	5.399727000	6.654193000
6	2.968785000	4.982839000	10.895503000
6	2.995227000	6.681106000	13.364493000
6	4.269561000	8.587666000	10.747898000
6	5.455760000	7.863316000	10.529315000
6	6.702047000	8.482832000	10.591750000
6	7.959365000	7.699878000	10.315624000
6	6.766838000	9.855200000	10.870716000
6	5.612071000	10.607585000	11.083447000
6	5.688612000	12.085082000	11.369057000
6	4.370876000	9.961275000	11.031732000
7	3.228620000	8.813210000	7.254666000
6	2.789711000	11.030698000	6.361920000
6	3.069123000	10.740409000	5.012488000
6	2.499565000	12.357986000	6.730268000
6	3.066534000	11.761893000	4.069058000
6	2.506377000	13.368308000	5.776292000
6	2.793513000	13.079642000	4.439981000
6	4.476960000	8.497011000	6.721655000
6	5.521112000	9.428576000	6.574921000
6	4.703365000	7.153134000	6.374049000
6	6.755065000	9.013818000	6.085801000
6	5.940799000	6.754480000	5.884107000
6	6.974936000	7.680962000	5.733431000

Supplementary Table 6. Computed Data for int-C

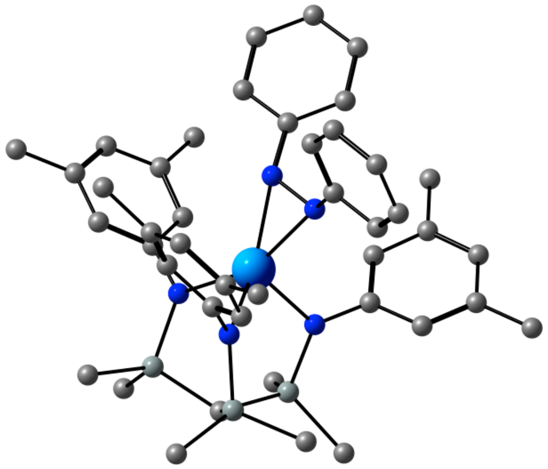

Temperature 298.150 Kelvin. Pressure 1.00000 Atm.

Zero-point correction= 0.886358 (Hartree/Particle)
Thermal correction to Energy= 0.947452
Thermal correction to Enthalpy= 0.948396
Thermal correction to Gibbs Free Energy= 0.781604
Sum of electronic and zero-point Energies= -2433.555212
Sum of electronic and thermal Energies= -2433.494119
Sum of electronic and thermal Enthalpies= -2433.493174
Sum of electronic and thermal Free Energies= -2433.659966

Charge = 0 Multiplicity = 2

92	1.799509000	8.749862000	8.904681000
14	-0.222122000	9.347223000	11.634197000
14	-0.530637000	6.539514000	10.166264000
14	2.089699000	7.125205000	11.900814000
7	0.208603000	9.813551000	9.977711000
7	0.594863000	6.906707000	8.840284000
7	2.936778000	8.095252000	10.667708000
7	3.126597000	10.304959000	8.101116000
6	0.218094000	7.482411000	11.657399000
6	0.730566000	10.395324000	12.894964000
6	-2.048809000	9.680427000	12.010558000
6	-0.322632000	10.842043000	9.183485000
6	-0.295818000	12.197625000	9.560551000
6	-0.762384000	13.189455000	8.702723000
6	-0.688734000	14.640581000	9.097757000
6	-1.256280000	12.824103000	7.440764000
6	-1.289170000	11.494082000	7.029051000
6	-1.805144000	11.112222000	5.666734000
6	-0.834933000	10.508421000	7.916301000
6	-2.295113000	7.066318000	9.710416000
6	-0.655685000	4.674391000	10.474901000

6	0.736929000	6.197128000	7.634829000
6	-0.273547000	6.176003000	6.651427000
6	-0.091559000	5.506594000	5.443100000
6	-1.189826000	5.459087000	4.412471000
6	1.132085000	4.864494000	5.200888000
6	2.160346000	4.876526000	6.143434000
6	3.482935000	4.215652000	5.858530000
6	1.946608000	5.537487000	7.359322000
6	2.582178000	5.307172000	11.679710000
6	2.625435000	7.544651000	13.671127000
6	4.344564000	8.238273000	10.702312000
6	5.148984000	7.546047000	9.787523000
6	6.537506000	7.728495000	9.750759000
6	7.369175000	6.997749000	8.729156000
6	7.121651000	8.599332000	10.668866000
6	6.348071000	9.295433000	11.609408000
6	7.008009000	10.227002000	12.593266000
6	4.967857000	9.111709000	11.613098000
7	2.834827000	9.389049000	7.070555000
6	3.157109000	11.675974000	7.847018000
6	2.896493000	12.236429000	6.585128000
6	3.490414000	12.526184000	8.917551000
6	2.957861000	13.616304000	6.412612000
6	3.544615000	13.901339000	8.730360000
6	3.280183000	14.458958000	7.476511000
6	3.805812000	9.083295000	6.118187000
6	5.105693000	9.618839000	6.147558000
6	3.451046000	8.204548000	5.077432000
6	6.023829000	9.268338000	5.161233000
6	4.379794000	7.864546000	4.101127000
6	5.672860000	8.393325000	4.132881000

Supplementary Table 7. Computed Data for int-D

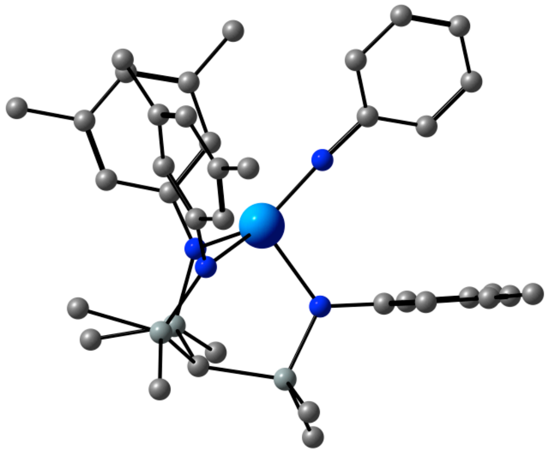

Temperature   298.150 Kelvin. Pressure   1.00000 Atm.

Zero-point correction=   0.790037 (Hartree/Particle)
Thermal correction to Energy=   0.845112
Thermal correction to Enthalpy=   0.846056
Thermal correction to Gibbs Free Energy=   0.692525
Sum of electronic and zero-point Energies=   -2147.373094
Sum of electronic and thermal Energies=   -2147.318019
Sum of electronic and thermal Enthalpies=   -2147.317075
Sum of electronic and thermal Free Energies=   -2147.470606

Charge = 0 Multiplicity = 2

92	2.350915000	11.672146000	3.802052000
14	4.821713000	11.437165000	1.392953000
14	4.918538000	13.875628000	3.498310000
14	2.729295000	13.853398000	1.125170000
7	3.975752000	10.634865000	2.715960000
7	3.593335000	13.332640000	4.554390000
7	1.616971000	12.796476000	2.002381000
7	1.428120000	11.080078000	5.426282000
6	4.464358000	13.293907000	1.719821000
6	4.173484000	10.753669000	-0.254481000
6	6.681036000	11.067393000	1.342139000
6	3.715784000	9.269783000	2.913852000
6	2.519830000	8.706599000	2.427911000
6	2.159666000	7.384823000	2.732848000
6	3.020299000	6.624963000	3.521727000
6	4.225217000	7.152086000	4.014631000
6	5.119873000	6.308517000	4.884950000
6	4.557469000	8.469390000	3.713197000
6	6.560321000	13.168201000	4.132358000
6	5.155148000	15.755497000	3.562027000
6	3.495483000	13.868137000	5.863412000
6	4.197668000	13.296601000	6.937300000
6	4.081076000	13.803141000	8.233228000

6	4.805274000	13.143638000	9.378403000
6	3.254371000	14.910222000	8.453357000
6	2.542900000	15.504573000	7.406767000
6	1.634067000	16.680373000	7.659917000
6	2.673770000	14.977300000	6.119149000
6	2.277214000	15.637362000	1.577089000
6	2.593509000	13.815525000	-0.768795000
6	0.440161000	12.134294000	1.696213000
6	-0.471862000	11.896489000	2.749835000
6	-1.598710000	11.071394000	2.584711000
6	-2.540233000	10.846846000	3.737521000
6	-1.836985000	10.515546000	1.333004000
6	-0.979113000	10.770411000	0.245380000
6	-1.291460000	10.184127000	-1.106998000
6	0.150334000	11.555678000	0.435232000
6	0.824336000	10.713568000	6.610281000
6	0.748557000	11.609100000	7.697758000
6	0.124989000	11.229374000	8.881171000
6	-0.435257000	9.957527000	9.016894000
6	-0.361449000	9.061763000	7.948929000
6	0.259228000	9.429189000	6.759617000
6	0.890273000	6.795594000	2.175239000

Supplementary Table 8. Computed Data for 3

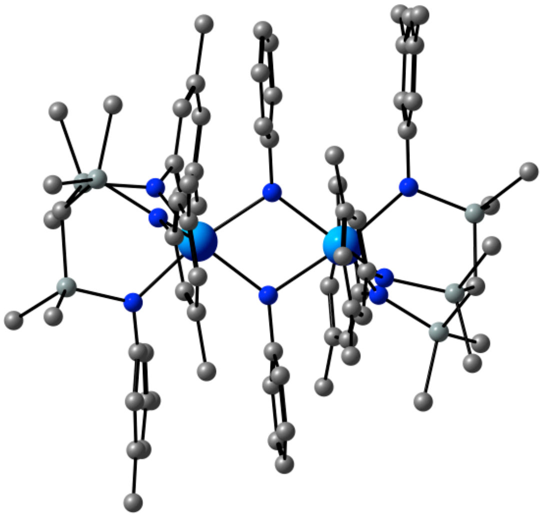

Temperature 298.150 Kelvin. Pressure 1.00000 Atm.

Zero-point correction= 1.584721 (Hartree/Particle)
Thermal correction to Energy= 1.694177
Thermal correction to Enthalpy= 1.695122
Thermal correction to Gibbs Free Energy= 1.429957
Sum of electronic and zero-point Energies= -4294.773860
Sum of electronic and thermal Energies= -4294.664405
Sum of electronic and thermal Enthalpies= -4294.663460
Sum of electronic and thermal Free Energies= -4294.928624

Charge = 0 Multiplicity = 3

92	1.824553000	9.500380000	7.524880000
14	0.057528000	9.821764000	10.506608000
14	-0.897123000	7.557620000	8.432055000
14	1.759168000	7.173519000	10.061390000
7	0.868228000	10.673296000	9.154092000
7	0.354067000	7.860691000	7.188845000
7	2.812984000	8.177563000	9.001351000
7	1.095675000	10.627873000	5.745479000
6	0.026739000	7.977004000	10.043609000
6	1.017970000	10.184919000	12.101899000
6	-1.703713000	10.437177000	10.868878000
6	1.185292000	12.009930000	9.513109000
6	2.506727000	12.364778000	9.822556000
6	2.829026000	13.638241000	10.303129000
6	4.256710000	13.991551000	10.626102000
6	1.803970000	14.566853000	10.484945000
6	0.476757000	14.254584000	10.168359000
6	-0.616437000	15.264126000	10.408978000
6	0.182556000	12.986093000	9.667631000
6	-2.451392000	8.586518000	8.105711000
6	-1.490955000	5.756878000	8.446048000

6	0.471613000	6.949181000	6.122772000
6	-0.612777000	6.634036000	5.277265000
6	-0.505752000	5.651887000	4.296032000
6	-1.691328000	5.286071000	3.439904000
6	0.712698000	4.973797000	4.141436000
6	1.814940000	5.276408000	4.941015000
6	3.102513000	4.504798000	4.818903000
6	1.686635000	6.276639000	5.913209000
6	1.780002000	5.366084000	9.482280000
6	2.458934000	7.068683000	11.821960000
6	4.197758000	7.977908000	9.252450000
6	4.882504000	6.885764000	8.691597000
6	6.219546000	6.629890000	9.000682000
6	6.941015000	5.458708000	8.384760000
6	6.883497000	7.490067000	9.880721000
6	6.238766000	8.596342000	10.440486000
6	6.977064000	9.535060000	11.357744000
6	4.898061000	8.825384000	10.123048000
6	-0.282273000	10.890909000	5.641316000
6	-1.127663000	10.074935000	4.866217000
6	-2.489762000	10.341263000	4.779603000
6	-3.049714000	11.424209000	5.459794000
6	-2.221180000	12.247029000	6.222782000
6	-0.855914000	11.987487000	6.315580000
92	2.830036000	11.337427000	4.571358000
14	4.891454000	11.321164000	1.770444000
14	5.020989000	13.851566000	3.721124000
14	2.555769000	13.444645000	1.830288000
7	4.330092000	10.400478000	3.210459000
7	3.719137000	13.362534000	4.858886000
7	1.754576000	12.178937000	2.825069000
7	3.554579000	10.348010000	6.427876000
6	4.424037000	13.136930000	2.059169000
6	4.144382000	10.565791000	0.197886000
6	6.764247000	11.167641000	1.490633000
6	4.739860000	9.041981000	3.100939000
6	3.901030000	8.088277000	2.506359000
6	4.342890000	6.786944000	2.241151000
6	3.449995000	5.821566000	1.505842000
6	5.636632000	6.427327000	2.621378000
6	6.488798000	7.342519000	3.253987000
6	7.875856000	6.926311000	3.672326000
6	6.034130000	8.640510000	3.481227000
6	6.699489000	13.202970000	4.305917000
6	5.215628000	15.731538000	3.564705000
6	3.224932000	14.328107000	5.758016000
6	4.076037000	15.099743000	6.574687000
6	3.584488000	16.141588000	7.359501000
6	4.518070000	17.010924000	8.162485000
6	2.207214000	16.394531000	7.367164000

6	1.327716000	15.618688000	6.612107000
6	-0.153479000	15.889760000	6.632372000
6	1.845905000	14.593497000	5.813300000
6	2.002092000	15.194647000	2.303238000
6	2.042564000	13.295775000	0.009393000
6	0.505908000	11.725003000	2.332273000
6	-0.634754000	12.544697000	2.373806000
6	-1.844231000	12.134432000	1.813587000
6	-3.058848000	13.021602000	1.888534000
6	-1.917889000	10.870955000	1.215267000
6	-0.811692000	10.019821000	1.178087000
6	-0.891754000	8.673362000	0.505571000
6	0.391824000	10.458329000	1.738748000
6	4.881430000	10.539343000	6.857496000
6	5.268152000	11.735057000	7.495963000
6	6.583601000	11.929935000	7.908199000
6	7.548751000	10.946698000	7.691273000
6	7.174868000	9.754508000	7.069717000
6	5.860732000	9.547041000	6.662688000

Supplementary Table 9. Computed Data for F

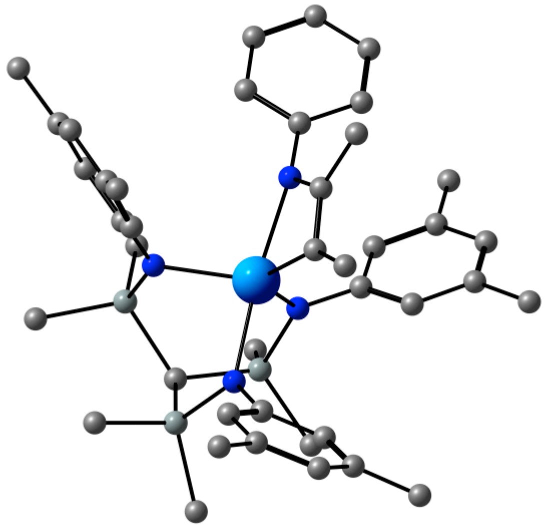

Temperature   298.150 Kelvin. Pressure   1.00000 Atm.

Zero-point correction= 0.877782 (Hartree/Particle)
Thermal correction to Energy= 0.938724
Thermal correction to Enthalpy= 0.939668
Thermal correction to Gibbs Free Energy= 0.773324
Sum of electronic and zero-point Energies= -2303.249494
Sum of electronic and thermal Energies= -2303.188553
Sum of electronic and thermal Enthalpies= -2303.187609
Sum of electronic and thermal Free Energies= -2303.353953

Charge = 0 Multiplicity = 2

92	-0.127010000	-0.187595000	-0.066700000
14	1.993145000	-0.058193000	-2.749143000
14	2.134394000	2.338119000	-0.635387000
14	-0.365028000	2.052665000	-2.671488000
7	1.490692000	-1.007132000	-1.330352000
7	1.100497000	1.530210000	0.562016000
7	-1.106903000	0.645667000	-1.885856000
7	-1.786543000	0.087565000	1.410160000
6	1.498351000	1.750333000	-2.341229000
6	1.183153000	-0.756227000	-4.316658000
6	3.855383000	-0.223070000	-3.073390000
6	2.040137000	-2.285336000	-1.081664000
6	1.594609000	-3.423645000	-1.776399000
6	2.126482000	-4.689092000	-1.517646000
6	3.114712000	-4.818068000	-0.535914000
6	3.580667000	-3.708137000	0.175728000
6	4.670749000	-3.858299000	1.205579000
6	3.037574000	-2.452385000	-0.105937000
6	3.940268000	1.885349000	-0.269922000
6	2.093047000	4.231122000	-0.498393000

6	1.255917000	1.746168000	1.947853000
6	1.814700000	0.738431000	2.756294000
6	1.927104000	0.893254000	4.140993000
6	2.559613000	-0.185112000	4.982690000
6	1.485170000	2.085995000	4.720581000
6	0.932866000	3.111301000	3.944752000
6	0.436710000	4.383072000	4.582992000
6	0.817230000	2.928543000	2.565794000
6	-1.033819000	3.675709000	-1.950299000
6	-0.754661000	2.142789000	-4.524241000
6	-2.340453000	0.046556000	-2.139431000
6	-3.525345000	0.767103000	-2.404991000
6	-4.743497000	0.116594000	-2.574819000
6	-5.998515000	0.894144000	-2.876631000
6	-4.796568000	-1.281722000	-2.454304000
6	-3.648998000	-2.028195000	-2.197864000
6	-3.701537000	-3.531184000	-2.109782000
6	-2.425828000	-1.356803000	-2.063500000
6	-2.575783000	1.043426000	2.063874000
6	-3.523906000	1.766744000	1.323813000
6	-4.292419000	2.752262000	1.936736000
6	-4.135639000	3.031616000	3.294359000
6	-3.188990000	2.321370000	4.033025000
6	-2.407243000	1.342134000	3.426430000
6	1.658919000	-5.891002000	-2.297571000
6	-1.574645000	-1.209054000	1.871189000
6	-0.581302000	-1.960120000	1.239904000
6	-2.448904000	-1.770190000	2.967864000
6	-0.301197000	-3.380087000	1.632815000

Supplementary Table 10. Computed Data for azobenzene

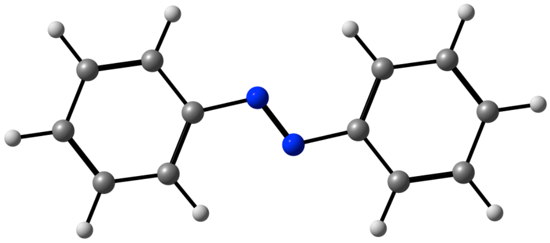

\-------------------

Temperature 298.150 Kelvin. Pressure 1.00000 Atm.

Zero-point correction= 0.191548 (Hartree/Particle)
Thermal correction to Energy= 0.202399
Thermal correction to Enthalpy= 0.203343
Thermal correction to Gibbs Free Energy= 0.153844
Sum of electronic and zero-point Energies= -572.353365
Sum of electronic and thermal Energies= -572.342514
Sum of electronic and thermal Enthalpies= -572.341570
Sum of electronic and thermal Free Energies= -572.391069

\-------------------

7	-0.004770000	0.628478000	0.000000000
7	0.004770000	-0.628478000	0.000000000
6	1.275317000	1.228729000	0.000000000
6	2.488291000	0.522578000	0.000000000
6	1.281929000	2.628067000	0.000000000
6	3.687634000	1.221134000	0.000000000
1	2.459806000	-0.561727000	0.000000000
6	2.488291000	3.322641000	0.000000000
1	0.326862000	3.144915000	0.000000000
6	3.692421000	2.620174000	0.000000000
1	4.628738000	0.677514000	0.000000000
1	2.489380000	4.408919000	0.000000000
1	4.636314000	3.158490000	0.000000000
6	-1.275317000	-1.228729000	0.000000000
6	-2.488291000	-0.522578000	0.000000000
6	-1.281929000	-2.628067000	0.000000000
6	-3.687634000	-1.221134000	0.000000000
1	-2.459806000	0.561727000	0.000000000
6	-2.488291000	-3.322641000	0.000000000
1	-0.326862000	-3.144915000	0.000000000
6	-3.692421000	-2.620174000	0.000000000
1	-4.628738000	-0.677514000	0.000000000
1	-2.489380000	-4.408919000	0.000000000
1	-4.636314000	-3.158490000	0.000000000

Supplementary Table 11. Computed Data for 2-butyne

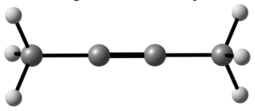

Temperature 298.150 Kelvin.
Pressure 1.00000 Atm.

Zero-point correction= 0.084369 (Hartree/Particle)
Thermal correction to Energy= 0.090121
Thermal correction to Enthalpy= 0.091065
Thermal correction to Gibbs Free Energy= 0.057520
Sum of electronic and zero-point Energies= -155.838279
Sum of electronic and thermal Energies= -155.832527
Sum of electronic and thermal Enthalpies= -155.831583
Sum of electronic and thermal Free Energies= -155.865128

6	0.000000000	0.000000000	2.062142000
1	0.000000000	1.021145000	2.460159000
1	0.884338000	-0.510573000	2.460159000
1	-0.884338000	-0.510573000	2.460159000
6	0.000000000	0.000000000	0.605134000
6	0.000000000	0.000000000	-0.605134000
6	0.000000000	0.000000000	-2.062142000
1	0.884338000	0.510573000	-2.460159000
1	0.000000000	-1.021145000	-2.460159000
1	-0.884338000	0.510573000	-2.460159000

Supplementary Table 12. Uranium-Large-Core Computed Data for int-D

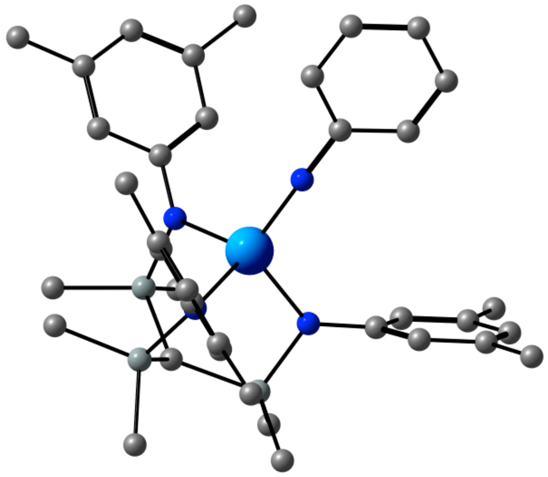

Temperature 298.150 Kelvin. Pressure 1.00000 Atm.

Zero-point correction= 0.790987 (Hartree/Particle)
Thermal correction to Energy= 0.846023
Thermal correction to Enthalpy= 0.846967
Thermal correction to Gibbs Free Energy= 0.691965
Sum of electronic and zero-point Energies= -1714.151361
Sum of electronic and thermal Energies= -1714.096325
Sum of electronic and thermal Enthalpies= -1714.095381
Sum of electronic and thermal Free Energies= -1714.250383

Charge = 0 Multiplicity = 1

92	2.420881000	11.599543000	3.912922000
14	4.646383000	11.108928000	1.578041000
14	4.764603000	13.819747000	3.470169000
14	2.350491000	13.462767000	1.241564000
7	3.941922000	10.270017000	2.980843000
7	3.576196000	13.329209000	4.700937000
7	1.265967000	12.430500000	2.206363000
7	1.280565000	10.697724000	5.239270000
6	4.124865000	12.959972000	1.848619000
6	3.954006000	10.319904000	0.002809000
6	6.524679000	10.944608000	1.415636000
6	4.206251000	8.892679000	3.198925000
6	3.218559000	7.928371000	2.963384000
6	3.455025000	6.571068000	3.205518000
6	4.711161000	6.180776000	3.672684000
6	5.720948000	7.119525000	3.915621000
6	7.057770000	6.679812000	4.455032000
6	5.457423000	8.468223000	3.676105000
6	6.482756000	13.251140000	4.024817000
6	4.918826000	15.691827000	3.249892000
6	3.629590000	13.894638000	6.002324000
6	4.224862000	13.198394000	7.063307000

6	4.261816000	13.734842000	8.353322000
6	4.869979000	12.952257000	9.488064000
6	3.704363000	14.997131000	8.572785000
6	3.103565000	15.717427000	7.535333000
6	2.468225000	17.058098000	7.799688000
6	3.074733000	15.157965000	6.256368000
6	1.900426000	15.273880000	1.558379000
6	2.166477000	13.239399000	-0.628122000
6	-0.127744000	12.431888000	1.935734000
6	-1.012287000	13.179412000	2.724537000
6	-2.389444000	13.167293000	2.480846000
6	-3.318560000	14.000129000	3.325947000
6	-2.879066000	12.394231000	1.425603000
6	-2.021678000	11.638003000	0.618797000
6	-2.572805000	10.775411000	-0.487137000
6	-0.651192000	11.667762000	0.881197000
6	0.459474000	10.055147000	6.138221000
6	-0.939362000	10.044278000	5.962533000
6	-1.766893000	9.391025000	6.869483000
6	-1.230144000	8.730699000	7.975638000
6	0.153007000	8.733463000	8.160904000
6	0.988402000	9.383812000	7.258986000
6	2.362353000	5.558716000	2.979805000

Supplementary Table 13. Uranium-Large-Core Computed Data for E

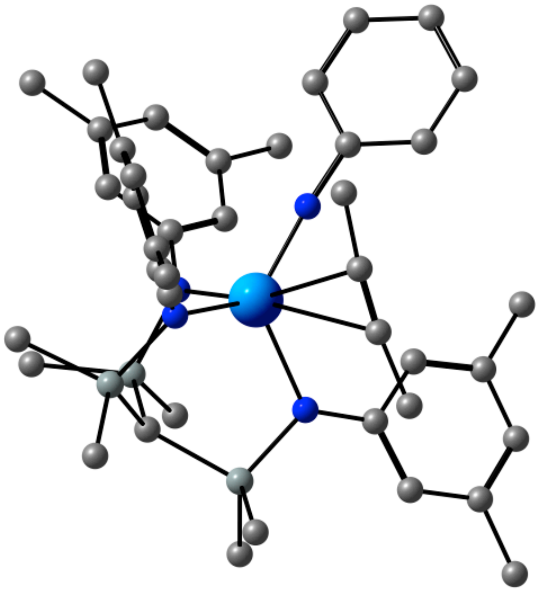

Temperature 298.150 Kelvin. Pressure 1.00000 Atm.

Zero-point correction= 0.877917 (Hartree/Particle)
Thermal correction to Energy= 0.939475
Thermal correction to Enthalpy= 0.940419
Thermal correction to Gibbs Free Energy= 0.775260
Sum of electronic and zero-point Energies= -1869.991440
Sum of electronic and thermal Energies= -1869.929882
Sum of electronic and thermal Enthalpies= -1869.928938
Sum of electronic and thermal Free Energies= -1870.094096

Charge = 0 Multiplicity = 1

92	0.136727000	-0.137288000	0.123850000
14	2.354062000	-0.834660000	-2.231902000
14	2.704057000	1.936908000	-0.365320000
14	0.391402000	1.748762000	-2.584630000
7	1.428674000	-1.627448000	-0.939023000
7	1.366039000	1.673764000	0.783890000
7	-0.780774000	0.792937000	-1.643728000
7	-1.524235000	-0.667603000	1.036576000
6	2.085788000	1.062725000	-1.973641000
6	1.708806000	-1.458612000	-3.898634000
6	4.200691000	-1.259768000	-2.230442000
6	1.366039000	-3.042265000	-0.928421000
6	0.205728000	-3.710493000	-1.350185000
6	0.109469000	-5.104626000	-1.303397000
6	1.200757000	-5.838229000	-0.829680000
6	2.375448000	-5.203954000	-0.410522000
6	3.550840000	-6.012107000	0.075950000
6	2.444984000	-3.810000000	-0.461057000
6	4.339897000	1.199332000	0.253701000

6	3.071722000	3.767655000	-0.724628000
6	1.110713000	2.502251000	1.887303000
6	2.081875000	3.339030000	2.464407000
6	1.795197000	4.146007000	3.571075000
6	2.856377000	5.054526000	4.137073000
6	0.517542000	4.106406000	4.129306000
6	-0.475403000	3.277710000	3.590950000
6	-1.860580000	3.255963000	4.183464000
6	-0.168769000	2.493230000	2.481725000
6	0.096851000	3.579368000	-2.203477000
6	0.185758000	1.605933000	-4.461740000
6	-2.160806000	0.935124000	-1.952854000
6	-2.984411000	1.776651000	-1.195025000
6	-4.346998000	1.903330000	-1.477571000
6	-5.226385000	2.764904000	-0.609406000
6	-4.880329000	1.188896000	-2.553552000
6	-4.081247000	0.349904000	-3.337272000
6	-4.666812000	-0.388486000	-4.513478000
6	-2.725260000	0.229136000	-3.025458000
6	-2.683238000	-0.912246000	1.731195000
6	-3.887006000	-1.203995000	1.053688000
6	-5.060573000	-1.445853000	1.758663000
6	-5.077387000	-1.413821000	3.154995000
6	-3.895471000	-1.128935000	3.838448000
6	-2.715574000	-0.877023000	3.142838000
6	-1.140390000	-5.798184000	-1.780485000
6	0.881580000	-2.119998000	2.561731000
6	1.686807000	-1.239023000	2.806884000
6	-0.019152000	-3.260531000	2.423139000
6	2.707345000	-0.315055000	3.294333000

Supplementary Table 14. Uranium-Large-Core Computed Data for TS_{FE}

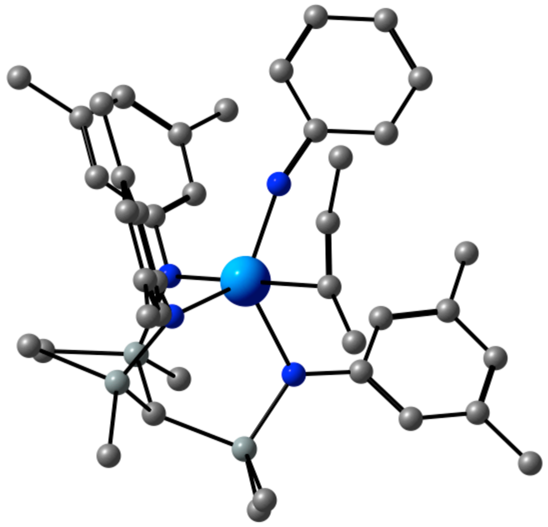

Temperature 298.150 Kelvin. Pressure 1.00000 Atm.

Zero-point correction= 0.877498 (Hartree/Particle)
Thermal correction to Energy= 0.937812
Thermal correction to Enthalpy= 0.938756
Thermal correction to Gibbs Free Energy= 0.776225
Sum of electronic and zero-point Energies= -1869.970583
Sum of electronic and thermal Energies= -1869.910270
Sum of electronic and thermal Enthalpies= -1869.909326
Sum of electronic and thermal Free Energies= -1870.071856

Charge = 0 Multiplicity = 1

92	2.555054000	11.597859000	4.211369000
14	4.753765000	11.087892000	1.714177000
14	5.087855000	13.778721000	3.595506000
14	2.724943000	13.586178000	1.443841000
7	3.897804000	10.273128000	3.055430000
7	3.738069000	13.452061000	4.701766000
7	1.646676000	12.556309000	2.401388000
7	0.956541000	10.731834000	5.132075000
6	4.476600000	12.977158000	1.959108000
6	4.046887000	10.409543000	0.094751000
6	6.608066000	10.696496000	1.636317000
6	3.891044000	8.856397000	3.102712000
6	2.695600000	8.144329000	2.916440000
6	2.656061000	6.750193000	2.997092000
6	3.840418000	6.056650000	3.261775000
6	5.049779000	6.733808000	3.444342000
6	6.323269000	5.970493000	3.704003000
6	5.061942000	8.128672000	3.368505000
6	6.731565000	13.039561000	4.198703000
6	5.442301000	15.628811000	3.404203000

6	3.476957000	14.140726000	5.895510000
6	4.483519000	14.506724000	6.810748000
6	4.178428000	15.147634000	8.009410000
6	5.268049000	15.522905000	8.980710000
6	2.838339000	15.423723000	8.314642000
6	1.813732000	15.078407000	7.434184000
6	0.377423000	15.412598000	7.742228000
6	2.146467000	14.447607000	6.229148000
6	2.360363000	15.404962000	1.838598000
6	2.442636000	13.450172000	-0.426061000
6	0.262991000	12.520008000	2.092288000
6	-0.637368000	13.409348000	2.696352000
6	-2.008140000	13.357261000	2.424117000
6	-2.959753000	14.286622000	3.131680000
6	-2.477592000	12.406151000	1.516076000
6	-1.605811000	11.508388000	0.888811000
6	-2.126342000	10.511422000	-0.114710000
6	-0.244198000	11.572426000	1.187539000
6	-0.219555000	10.858335000	5.853858000
6	-1.430661000	10.432758000	5.272269000
6	-2.637769000	10.584028000	5.947011000
6	-2.674065000	11.138838000	7.227277000
6	-1.481136000	11.552335000	7.821483000
6	-0.270163000	11.422251000	7.146870000
6	1.365275000	6.007757000	2.763970000
6	2.472378000	9.878733000	6.349629000
6	3.582053000	10.484507000	6.210316000
6	1.689871000	8.765793000	6.906514000
6	4.971228000	10.380094000	6.737472000

Supplementary Table 15. Uranium-Large-Core Computed Data for F

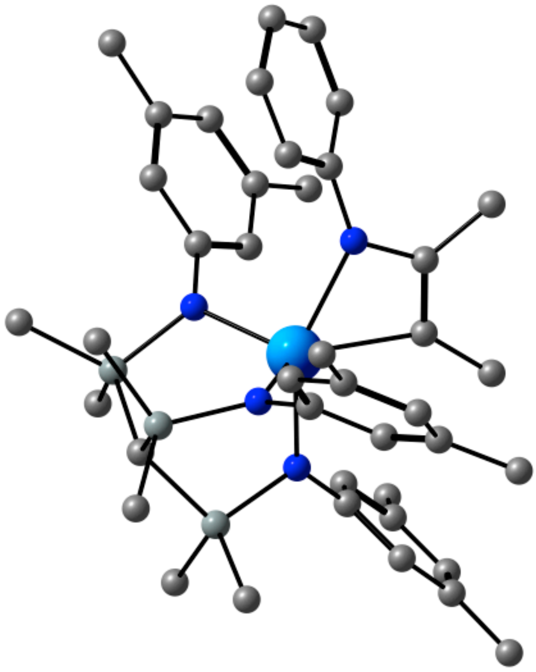

Temperature 298.150 Kelvin. Pressure 1.00000 Atm.

Zero-point correction= 0.878644 (Hartree/Particle)
Thermal correction to Energy= 0.939562
Thermal correction to Enthalpy= 0.940507
Thermal correction to Gibbs Free Energy= 0.772347
Sum of electronic and zero-point Energies= -1870.015606
Sum of electronic and thermal Energies= -1869.954687
Sum of electronic and thermal Enthalpies= -1869.953743
Sum of electronic and thermal Free Energies= -1870.121903

Charge = 0 Multiplicity = 1

92	-0.285544000	-0.039059000	-0.058907000
14	1.866533000	-0.195950000	-2.553443000
14	1.946373000	2.391057000	-0.585221000
14	-0.528846000	1.946594000	-2.722169000
7	1.353366000	-1.124121000	-1.105701000
7	0.943293000	1.626260000	0.673748000
7	-1.345356000	0.593686000	-1.899299000
7	-1.877238000	0.009230000	1.553592000
6	1.307802000	1.629709000	-2.234039000
6	1.102310000	-0.942337000	-4.118191000
6	3.737371000	-0.306506000	-2.822304000
6	2.027250000	-2.354698000	-0.901676000
6	1.691986000	-3.502498000	-1.632352000
6	2.352521000	-4.717432000	-1.422381000
6	3.361036000	-4.778659000	-0.458521000

6	3.722431000	-3.649951000	0.286349000
6	4.834764000	-3.726927000	1.300335000
6	3.051594000	-2.448604000	0.053696000
6	3.760086000	1.999872000	-0.211262000
6	1.842798000	4.282539000	-0.597109000
6	1.242624000	1.837269000	2.045509000
6	1.823496000	0.813282000	2.810642000
6	2.083170000	0.984533000	4.172685000
6	2.735631000	-0.113599000	4.972034000
6	1.761656000	2.208096000	4.767998000
6	1.182966000	3.245968000	4.031893000
6	0.791647000	4.540747000	4.695235000
6	0.927464000	3.049238000	2.672748000
6	-1.190280000	3.622576000	-2.130381000
6	-0.792372000	1.929591000	-4.595542000
6	-2.566718000	0.011178000	-2.286907000
6	-3.688268000	0.776818000	-2.654425000
6	-4.895386000	0.172240000	-2.999490000
6	-6.082815000	1.002873000	-3.412633000
6	-4.993360000	-1.225067000	-2.957178000
6	-3.904578000	-2.014292000	-2.588101000
6	-4.020099000	-3.514841000	-2.523272000
6	-2.694184000	-1.386730000	-2.271882000
6	-2.477493000	1.004055000	2.327236000
6	-3.278710000	1.966029000	1.683413000
6	-3.864508000	3.005471000	2.399299000
6	-3.684425000	3.104538000	3.779136000
6	-2.891336000	2.157473000	4.428248000
6	-2.284188000	1.127485000	3.716094000
6	1.987040000	-5.933064000	-2.234988000
6	-1.705508000	-1.345022000	1.940796000
6	-0.769776000	-1.979694000	1.170972000
6	-2.591469000	-1.941047000	3.003724000
6	-0.444373000	-3.441087000	1.235322000

Supplementary Methods

General Experimental Details

All manipulations were carried out using Schlenk techniques, or an MBraun UniLab glovebox, under an atmosphere of dry nitrogen. Solvents were dried by passage through activated alumina towers, degassed before use, and were stored over potassium mirrors. Deuterated solvent was distilled from potassium, degassed by three freeze-pump-thaw cycles and stored under nitrogen. Potassium graphite[6] and [U(Ts^{Xy})(Cl)(THF)] (**1**)[7] were prepared as previously described. Azobenzene and alkynes were dried under vacuum for four hours prior to use or were made up as standard solutions in toluene that were dried over 4 Å sieves. D_{10}-azobenzene was prepared as described previously.[8] Tmeda and pmdeta were distilled from CaH_2 prior to use. Acetonitrile-d_3, Nujol®, methanol (analytical grade for mass spectrometry), and PhN_3 (anhydrous 0.5 M solution in Bu^{t}OMe) were purchased from Sigma-Aldrich and used as received.

^{1}H NMR spectra were recorded at 298 K on Bruker DPX 300 or AV(III)400 spectrometers operating at 300.1 or 400.0 MHz, respectively; chemical shifts are quoted in ppm and are relative to TMS. FTIR spectra were recorded on a Bruker Tensor 27 spectrometer. UV/Vis/NIR spectra were recorded on a Perkin Elmer Lambda 750 spectrometer; data were collected in 1mm path length cuvettes and were run versus the appropriate reference solvent. Variable-temperature magnetic moment data were recorded in an applied dc field of 0.1 T on a Quantum Design MPMS XL7 superconducting quantum interference device (SQUID) magnetometer using doubly recrystallised powdered samples. Samples were carefully checked for purity and data reproducibility between independently prepared batches for each compound examined. Care was taken to ensure complete thermalisation of the sample before each data point was measured and samples were immobilised in an eicosane matrix to prevent sample reorientation during measurements. Diamagnetic corrections were applied using tabulated Pascal constants and measurements were corrected for the effect of the blank sample holders and eicosane matrix. Solution magnetic moments were recorded at room

temperature using the Evans method. Discrepancies between the solution and solid state magnetic moments are attributed to the differences in phase of these measurements; we note that SQUID magnetometry is the more sensitive and accurate of the two methods. Variable temperature (300-5 K) EPR spectra were measured at X-band (ca. 9.4 GHz) on a Bruker EMX spectrometer. Polycrystalline samples were sealed under vacuum in 1mm i.d. silica tubing, and double-contained for EPR by insertion into an X-band silica tube or PTFE sleeve. Measurements were made on independently prepared batches, which were also analysed by SQUID and CHN microanalyses, to ensure reproducibility. Spectra were background corrected against blank sample holders measured under identical conditions. Mass spectrometry was carried out using a Bruker MicroTOF (ESI-MS) instrument. CHN microanalyses were carried out by Tong Liu at the University of Nottingham or Martin Jennings at the University of Manchester. Cyclic voltammetry experiments could not be performed on any of the uranium complexes reported here because of the onset of decomposition in polar solvents and reactions with electrolytes.

General Computational Details

All the structures reported in this study were fully optimised with the Becke's 3-parameter hybrid functional combined with the non-local correlation functional provided by Perdew/Wang.[9,10] The Stuttgart-Cologne relativistic energy-consistent small-core pseudopotential is used for U atom, in combination with its adapted segmented basis set.[11,12] For Si atom the corresponding effective core potential (ECP) is chosen in combination with its adapted basis sets,[13] augmented by an extra set of polarisation functions.[14] For the rest of the atoms the 6-31G(d,p) basis set is used.[15-17] The aforementioned computational protocol is found to be adequate for this study as it reproduce finely the geometry of complex **3** (see Figure S23). In contrast, for the insertion reaction presented in Figure S26, where there is no change in oxidation state of the uranium, being +5, the corresponding ECP and its associated basis set is used,[18] augmented by a f polarization function ($\alpha = 1.00$). This corresponds to the Stuttgart-Cologne's quasi-relativistic energy-consistent 5*f*-in-core

pseudopotential basis set (large-core), and chosen for its computational efficacy. In all computations no constrains were imposed on the geometry. All stationary points have been identified for minimum (number of imaginary frequencies Nimag=0) or transition states (Nimag=1). Intrinsic Reaction Paths (IRPs) are traced from the various transition structures to verify the reactant to product linkage.[19,20] The GAUSSIAN09 program suite is used in all calculations.[21]

Supplementary References

1. Lam, O. P., Heinemann, F. W. & Meyer, K. Activation of elemental S, Se, and Te with uranium(III): bridging U-E-U (E = S, Se) and diamond core complexes U-$(E)_2$-U (E = O, S, Se, Te). *Chem. Sci.* **2**, 1538-1547 (2011).

2. Arnold, P. L., Jones, G. M., Odoh, S. O., Schreckenbach, G., Magnani, N. & Love, J. B. Strongly coupled binuclear uranium-oxo complexes from uranyl oxo rearrangement and reductive silylation. *Nat. Chem.* **4**, 221-227 (2012).

3. Nocton, G, Horeglad, P., Pécaut, J. & Mazzanti, M. Polynuclear cation-cation complexes of pentavalent uranyl: relating stability and magnetic properties to structure. *J. Am. Chem. Soc.* **130**, 16633-16645 (2008).

4. Rosen, R. K., Andersen, R. A. & Edelstein, N. M. $[(MeC_5H_4)_3U]_2[\mu\text{-}1,4\text{-}N_2C_6H_4]$: a bimetallic molecule with antiferromagnetic coupling between the uranium centers *J. Am. Chem. Soc.* **112**, 4588-4590 (1990).

5. King, D. M., Tuna, F., McInnes, E. J. L., McMaster, J., Lewis, W., Blake, A. J. & Liddle, S. T. Synthesis and structure of a terminal uranium nitride complex. *Science* **337**, 717-720 (2012).

6. Bergbreiter, D. E. & Killough, J. M. Reactions of potassium-graphite. *J. Am. Chem. Soc.* **100**, 2126-2134 (1978).

7. Patel, D., Lewis, W., Blake, A. J. & Liddle, S. T. Uranium(IV) amide and halide derivatives of two tripodal *tris*(*N*-arylamidodimethylsilyl)methanes. *Dalton Trans.* **39**, 6638-6647 (2010).

8. Stern, M. K., Cheng, B. K., Hileman, F. D. & Allman, J. M. A new route to 4-aminodiphenylamine via nucleophilic aromatic substitution for hydrogen: reaction of aniline and azobenzene. *J. Org. Chem.* **59**, 5627-5632 (1994).

9. Becke, A. D. Density-functional thermochemistry. III. The role of exact exchange. *J. Chem. Phys*. **98**, 5648-5652 (1993).

10. Perdew, J. P. & Wang, Y. Accurate and simple analytic representation of the electron-gas correlation energy. *Phys. Rev. B* **45**, 13244 (1992).

11. Kuchle, W. Dolg, M., Stoll, H. & Preuss, H. Energy-adjusted pseudopotentials for the actinides. Parameter sets and test calculations for thorium and uranium monoxide. *J. Chem. Phys*. **100**, 7535-7542 (1994).

12. Cao, X. Y., Dolg, M. & Stoll, H. Valence basis sets for relativistic energy-consistent small-core actinide pseudopotentials. *J. Chem. Phys*. **118**, 487-496 (2003).

13. Bergner, A., Dolg, M., Kuechle, W., Stoll, H. & Preuss, H. Ab initio energy-adjusted pseudopotentials for elements of groups 13-17. *Mol. Phys.* **80**, 1431-1441 (1993).

14. Ehlers, A. W., Böhme, M., Dapprich, S., Gobbi, A., Höllwarth, A., Jonas, V., Köhler, K. F., Stegmann, R., Veldkamp, A. & Frenking, G. A set of f-polarization functions for pseudo-potential basis sets of the transition metals ScCu, YAg and LaAu. *Chem. Phys. Lett.* **208**, 111-114 (1993).

15. Ditchfield, R., Hehre, W. J. & Pople, J. A. Self-consistent molecular-orbital methods. IX. An extended Gaussian-type basis for molecular-orbital studies of organic molecules. *J. Chem. Phys.* **54**, 724-728 (1971).

16. Hehre, W. J., Ditchfield, R. & Pople, J. A. Self-consistent molecular orbital methods. XII. Further extensions of Gaussian-type basis sets for use in molecular orbital studies of organic molecules. *J. Chem. Phys.* **56**, 2257-2262 (1972).

17. Hariharan, P. C. & Pople, J. A. The influence of polarization functions on molecular orbital hydrogenation energies. *Theor. Chim. Acta.* **28**, 213-222 (1973).

18. A. Moritz, M. Dolg, Quasirelativistic energy-consistent 5f-in-core pseudopotentials for pentavalent and hexavalent actinide elements. *Theor. Chem. Acc.* **121**, 297-306 (2008).

19. Gonzalez, C. & Schlegel, H. B. An improved algorithm for reaction path following. *J. Chem. Phys.* **90**, 2154-2162 (1989).

20. Gonzalez, C. & Schlegel, H. B. Reaction path following in mass-weighted internal coordinates. *J. Phys. Chem.* **94**, 5523-5527 (1990).

21. Gaussian 09, Revision D.01, Frisch, M. J., Trucks, G. W., Schlegel, H. B., Scuseria, G. E., Robb, M. A., Cheeseman, J. R., Scalmani, G., Barone, V., Mennucci, B., Petersson, G. A., Nakatsuji, H., Caricato, M., Li, X., Hratchian, H. P., Izmaylov, A. F., Bloino, J., Zheng, G., Sonnenberg, J. L., Hada, M., Ehara, M., Toyota, K., Fukuda, R., Hasegawa, J., Ishida, M., Nakajima, T., Honda, Y., Kitao, O., Nakai, H., Vreven, T., Montgomery, J. A., Jr., Peralta, J. E., Ogliaro, F., Bearpark, M., Heyd, J. J., Brothers, E., Kudin, K. N., Staroverov, V. N., Kobayashi, R., Normand, J., Raghavachari, K., Rendell, A., Burant, J. C., Iyengar, S. S., Tomasi, J., Cossi, M., Rega, N., Millam, J. M., Klene, M., Knox, J. E., Cross, J. B., Bakken, V., Adamo, C., Jaramillo, J., Gomperts, R., Stratmann, R. E., Yazyev, O., Austin, A. J., Cammi, R., Pomelli, C., Ochterski, J. W., Martin, R. L., Morokuma, K., Zakrzewski, V. G., Voth, G. A., Salvador, P., Dannenberg, J. J., Dapprich, S., Daniels, A. D., Farkas, Ö., Foresman, J. B., Ortiz, J. V., Cioslowski, J., Fox, D. J., Gaussian, Inc., Wallingford CT, 2013.
